# Production of monoclonal antibodies against GPCR using cell-free synthesized GPCR antigen and biotinylated liposome-based interaction assay

**DOI:** 10.1038/srep11333

**Published:** 2015-06-10

**Authors:** Hiroyuki Takeda, Tomio Ogasawara, Tatsuhiko Ozawa, Atsushi Muraguchi, Pei-Ju Jih, Ryo Morishita, Motokazu Uchigashima, Masahiko Watanabe, Toyoshi Fujimoto, Takahiro Iwasaki, Yaeta Endo, Tatsuya Sawasaki

**Affiliations:** 1 Proteo-Science Center, Ehime University, Ehime 791-8577, Japan; 2 Graduate School of Medicine and Pharmaceutical Sciences for Research, University of Toyama, Toyama 930-0194, Japan; 3 Abnova (Taiwan) Corporation, Taoyuan County 320, Taiwan; 4 Cell-Free Sciences Co., Ltd., Ehime 791-8577, Japan; 5 Graduate School of Medicine, Hokkaido University, Hokkaido 060-8638, Japan; 6 Graduate School of Medicine, Nagoya University, Aichi 466-8550, Japan; 7 Institute for the Promotion of Science and Technology, Ehime University, Ehime 791-8577, Japan

## Abstract

G-protein-coupled receptors (GPCRs) are one of the most important drug targets, and anti-GPCR monoclonal antibody (mAb) is an essential tool for functional analysis of GPCRs. However, it is very difficult to develop GPCR-specific mAbs due to difficulties in production of recombinant GPCR antigens, and lack of efficient mAb screening method. Here we describe a novel approach for the production of mAbs against GPCR using two original methods, bilayer-dialysis method and biotinylated liposome-based interaction assay (BiLIA), both of which are developed using wheat cell-free protein synthesis system and liposome technology. Using bilayer-dialysis method, various GPCRs were successfully synthesized with quality and quantity sufficient for immunization. For selection of specific mAb, we designed BiLIA that detects interaction between antibody and membrane protein on liposome. BiLIA prevented denaturation of GPCR, and then preferably selected conformation-sensitive antibodies. Using this approach, we successfully obtained mAbs against DRD1, GHSR, PTGER1 and T1R1. With respect to DRD1 mAb, 36 mouse mAbs and 6 rabbit mAbs were obtained which specifically recognized native DRD1 with high affinity. Among them, half of the mAbs were conformation-sensitive mAb, and two mAbs recognized extracellular loop 2 of DRD1. These results indicated that this approach is useful for GPCR mAb production.

GPCRs constitute the largest family of membrane proteins in human, which are responsible for most cellular responses to hormones and neurotransmitters as well as light sensing, olfaction, and taste^1^,^2^. GPCRs are thought to be one of the most important drug targets. Approximately half of the current drugs target GPCRs^3^. Researchers passionately conduct cell biological, biochemical and structural studies of GPCRs, aiming at GPCR-targeted drug discovery as well as antibody therapeutics^4^.

mAb against GPCR is essential for functional and structural analyses of GPCR in investigating the temporal and spatial expressions, stabilizing the structure as chaperone binder, as well as functional regulation of receptors^4^,^5^,^6^,^7^. However, high-quality mAbs are still difficult to develop because of the lack of suitable antigen. Currently there are several choices for immunizing and screening antigen, such as peptide, whole cell or membrane fraction of transfected cultured cell, DNA, virus-like particle, or purified protein reconstituted in proteoliposome^4^. However, each of them has limitations in immunogenicity, structure, quantity, stability or versatility. For instance, synthetic peptides of loop or terminal sequence of GPCR are the most widely used antigens; however, this kind of linear antigen would not represent the structural feature of native GPCR. Usually GPCRs are difficult to overexpress in cellular system, leading to low efficiency of DNA, whole cell or cell membrane immunization. Among the antigens mentioned above, proteoliposome is the most promising antigen because the highly concentrated and purified antigen receptor is stabilized on the lipid vesicle (liposome). The problem of proteoliposome antigen is the mass production of high quality GPCR protein^4^. *E. coli* system is not suitable for GPCR preparation, in which expressed membrane protein often form inclusion body^8^,^9^. Many reports have shown successful GPCR expression using baculovirus-infected insect cells or yeast systems^10^,^11^,^12^,^13^. Only a few stable GPCRs, however, are easily prepared in large quantity by these systems, and in many cases researchers still have to take the trouble to search for the specific optimal conditions on overexpression, solubilization, purification, and reconstitution for each GPCR of interest. As to the GPCRs that are too unstable to express, mutations such as amino acid substitution, insertion of T4 lysozyme (T4L) and amino-terminal b562RIL (BRIL) are often used to stabilize it^1^,^14^.

Novel assay method for membrane protein-antibody interaction is needed for development of conformation-sensitive antibodies against GPCR. The assay requires high sensitivity, high throughput and wide dynamic range. Minimization of GPCR antigen denaturation during the assay procedure is also necessary. Conventional ELISA does not meet these requirements sufficiently, in which antigen is immobilized on the solid state and exposed to air in each washing step inevitably. In-solution assay, in which structure of liposome is easily sustained, has advantage.

Last, cell-free system has received considerable attention as one of the promising alternative method instead of cellular system in recent years. Cell-free system is free from the control and effect of intracellular trafficking and signaling pathways, and thus it has the potential to produce variety of membrane proteins efficiently^15^. Indeed, reports have increased much lately on cell-free synthesis of membrane proteins, in which liposome or detergent is added into the reaction^15^,^16^,^17^,^18^,^19^,^20^,^21^. However, it is not practical enough to prepare mg of immunizing antigen using the existing cell-free methods considering their limited productivity. In this study, we propose a improved membrane protein synthesis method based on wheat cell-free system, which is sufficient enough to prepare mg of immunizing antigen with low cost and effort. We also developed a new antibody-membrane protein interaction assay using biotinylated proteoliposome and AlphaScreen technology^22^. In this paper, we describe our work on the production, selection and characterization of mAbs against several GPCR targets using these technologies, and discuss their practicality in antibody development.

## Results

### Production of GPCR antigen using bilayer-dialysis method

**Figure 1:**
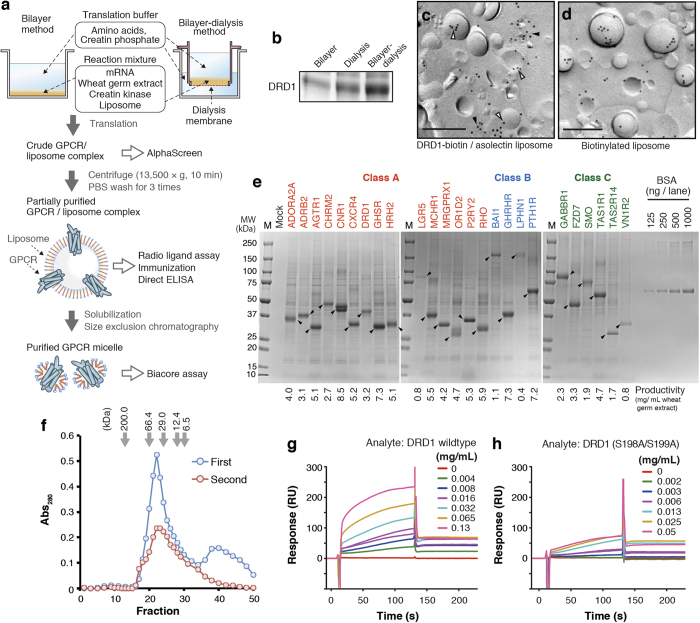
Production and purification of GPCRs using wheat cell-free synthesis system. (**a**) A schematic representation. Wheat cell-free synthesis of GPCR was conducted by bilayer method^15^ or bilayer-dialysis method. GPCR/liposome complex or solubilized GPCR micelle was prepared depending on the purposes. (**b**) Comparison of productivity between the synthesis methods. DRD1 was synthesized with each method. The ratio of wheat germ extract: liposome: mRNA was shared. DRD1 liposome was partially purified by centrifugation, and purified liposome containing 0.8 μg lipid was subjected to SDS-PAGE. CBB staining image is shown. (**c**) Immunogold labeling of DRD1-biotin/liposome complex. Bar, 0.2 μm. Blank arrowheads indicate DRD1-biotin located on liposome, and filled arrowheads present ones on lipid structures in close association with liposomes. (**d**) Immunogold labeling of biotinylated liposome. Bar, 0.2 μm. (**e**) SDS-PAGE image of cell-free synthesized GPCRs. Twenty five GPCRs were synthesized by bilayer-dialysis method with 100 μL reaction mixture containing 25 μL of wheat germ extract. GPCR/liposome complexes were partially purified by centrifugation and PBS, and then resuspended into 100 μL of PBS. Two μL liposome suspension was applied to SDS-PAGE and CBB staining. Arrowheads indicate target GPCRs. BSA standards were also applied. (**f**) SEC elution profile of solubilized DRD1. Partially purified DRD1 liposome was solubilized by DDM-containing buffer and subjected to SEC (first run, blue). DRD1 peak fractions in first run (fraction 18–25) were collected, concentrated by ultrafiltration, and applied to SEC (second run, red). (**g**, **h**) Biacore analysis of DRD1. Dopamine and histamine were immobilized on the measuring cell and reference cell of a sensor chip, respectively. DRD1 wild-type (**c**) or S198A/S199A mutant (**d**) micelle was injected as analyte.

To maximize the productivity of GPCR, we combined two existing cell-free protein synthesis methods, called bilayer-dialysis method. Reaction mixture containing wheat germ extract, mRNA and asolectin liposome was injected under the translation buffer in a cup-type dialysis device, which is immersed in the translation buffer (Fig. 1a). Supplement of substrates and purging of byproduct were conducted at both the top and bottom interfaces of reaction mixture, and the membrane protein was translated efficiently. In this study yield of synthesized DRD1 per wheat germ extract was approximately eight times and twice as much as that with bilayer method and dialysis method, respectively (Fig. 1b).

We confirmed the association between cell-free synthesized DRD1 and liposome by observing DRD1/liposome complex under electron microscopy. C-terminus biotinylated DRD1 was synthesized with bilayer method in the presence of liposome, biotin, and biotin ligase BirA^23^. The liposome fraction was collected by centrifugation and subjected to electron microscopy using freeze-fracture replica labeling technique. Some biotinylated DRD1 signals were located in liposomes (Fig. 1c, blank arrow heads), and their distribution pattern was similar to the signals of biotin-CAP DPPE in liposomes [1% (w/w) biotin-CAP DPPE in asolectin] (Fig. 1d). Meanwhile, biotinylated DRD1 signals were also observed on structures in close association with liposomes (Fig. 1c, filled arrow heads).

In order to determine the productivity of GPCRs with the present method, we randomly selected 25 GPCRs from different classes and families. Full-length ORF of them was sub-cloned into pEU-E01 vector^24^ respectively, and applied to cell-free synthesis system using bilayer-dialysis method. Synthesized GPCR/liposome complexes were partially purified by centrifugation and resuspension in phosphate-buffered saline (PBS) for three times, and visualized by SDS-PAGE and CBB staining (Fig. 1e). All of the GPCRs were synthesized and purified (60–70% purity) successfully. In this experiment, 25 μL of WEPRO7240 wheat germ extract was added into a translation reaction mixture, and 1/50 of the reaction mixture was loaded to the SDS-PAGE gel. The productivity of each cell-free synthesized GPCR was evaluated by the band intensity. Calibration curve was obtained with BSA standards. CNR1 showed the highest productivity (8.5 mg/mL wheat germ extract). Twenty-two out of 25 GPCRs showed productivity of more than 1 mg/mL extract, and 14 were more than 4 mg/mL extract. Even class B GPCR, containing large extra cellular domain, such as BAI1 (173.5 kDa) was observed as a clear band with productivity of 1.1 mg/mL extract. This measurement would be underestimated because CBB stainability of membrane protein is lower than BSA in general.

To evaluate the quality of GPCR synthesized by bilayer-dialysis method, ligand-binding activity of DRD1 was determined by surface plasmon resonance (SPR). DRD1 liposome was solubilized in *n*-dodecyl-β-d-maltopyranoside (DDM)-containing buffer and purified size-exclusion chromatography (SEC) (Fig. 1f). No aggregation peak was observed in void fraction. DRD1 was eluted in the main peak (fraction 19–30). Second peak was observed in the low-molecular weight range of the first SEC profile, but when the DRD1 peak was collected and subjected to SEC again, the second peak disappeared. SEC-purified DRD1 micelle (fraction 19-25) was used for Biacore assay as an analyte. Dopamine was immobilized on a CM5 sensor chip by amine coupling. In the reference cell, histamine was immobilized at the same level. DRD1 micelle specifically bound to dopamine at K_D_ value = 0.7 × 10^–6^ M (Fig. 1g). This value was agreed closely with radio ligand assay in our former report^25^. S198A/S199A, a ligand-binding deficient mutation^26^, canceled the binding (Fig. 1h). The result suggests that cell-free synthesized DRD1, at least in part, retained correct tertiary structure. Taken together, these results indicate that bilayer-dialysis method is very suitable for mass production of GPCR proteins.

### Development of biotinylated liposome-based interaction assay (BiLIA) for detection of antibody-membrane protein interaction on liposome

**Figure 2:**
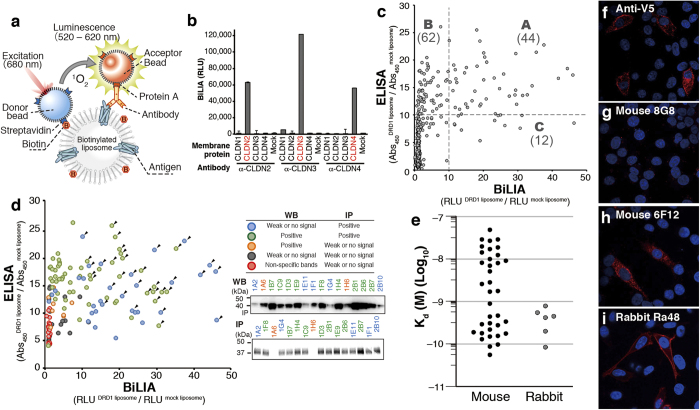
Development and characterization of anti-DRD1 antibodies using cell-free synthesized antigen. (**a**) Schematic diagram of antibody screening by BiLIA. GPCR antigen was synthesized on a biotinylated liposome. When antibody binds to GPCR as illustrated, AlphaScreen beads generate chemiluminescence signal. (**b**) Subtype specific binding of anti-claudin (CLDN) antibodies detected by BiLIA. CLDN2, 3, 4 were synthesized by bilayer method in the presence of biotinylated liposome. Anti-claudin antibodies were purchased from Life Technologies. Error bars represent standard deviation of the mean (n = 4). (**c**) Primary screening of mouse mAbs. Each culture supernatant of 764 hybridoma clones was assayed by both ELISA and BiLIA, respectively. Each plot represents a hybridoma clone. Broken lines indicate a threshold of specific binding. (**d**) Binding mode analysis of 143 mouse hybridoma clones. Supernatant of selected 143 clones was analyzed by WB and IP. Representative results of WB and IP were shown and detailed results of each supernatant were shown in Supplementary Table S2 online. Color of each plot indicates the binding mode. Arrowheads indicate 36 mAb clones selected for further analysis. (**e**) Affinity of 36 mouse mAbs and 6 rabbit mAbs. The antibody affinity (Kd) was determined by Scatchard plots. All data are representative out of at least two independent experiments with similar results. (**f**–**i**) Immunostaining of DRD1-V5 overexpressed HeLa cells. Representative data were shown. Red, antibody detected by PLA; blue, DAPI. (**f**) Anti-V5 antibody. (**g**) Mouse mAb clone 8G8, where significant PLA signal was not detected. (**h**) Mouse mAb clone 6F12. (I) Rabbit mAb clone Ra48.

To design high-throughput screening method for antibodies against GPCR, we used AlphaScreen technology for detection, because its homogeneous assay style and high sensitivity is suitable for high-throughput protein-antibody interaction assay. Previously we reported protein-autoantibody interaction method using AlphaScreen^27^. Biotinylated antigen is required for constructing AlphaScreen-based antibody-membrane protein interaction assay. We prepared biotinylated liposome by adding 1,2-dipalmitoyl-sn-glycero-3-phosphoethanolamine-N-(cap biotinyl) (biotin-CAP-DPPE) to base asolectin at 1% (w/w) concentration, and an antigen membrane protein was synthesized on it. It is an advantage of this approach that native-form membrane protein without artificial fusion of protein or peptide tag can be used as antigen. We mixed the protein/biotinylated liposome complex with antibody in a 384-well titer plate, and then added AlphaScreen beads. When antibody bound to the protein on a biotinylated liposome, the complex (donor bead-liposome-antigen-antibody-acceptor bead, as illustrated in Fig. 2a) was formed, and chemiluminescence signal was observed. To check performance of this biotinylated liposome-based interaction assay (BiLIA), we synthesized claudin (CLDN) family having four transmembrane regions because antibodies against each claudin are commercially available. Indeed, BiLIA could detect specific binding between claudin 2, 3, 4 synthesized on biotinylated liposome and corresponding anti-claudin antibodies, respectively (Fig. 2b). These results demonstrated that BiLIA using biotinylated liposome and AlphaScreen could specifically detect antibody-membrane protein interaction on liposome.

### Production of mouse mAbs against DRD1

We produced mouse mAbs and evaluated the resultant antibodies. One mg of DRD1 protein was synthesized by bilayer-dialysis method, purified by centrifuge, and immunized to mice. Hybridoma cells were then generated by fusing immune spleen cells with myeloma cell, and 764 hybridoma clones were isolated.

In the primary screening, mouse anti-DRD1 mAbs were investigated using conventional Enzyme-Linked Immunosorbent Assay (ELISA) and BiLIA (Fig. 2c and Supplementary Table S2 online). Culture supernatant from each clone was collected and reacted with both biotinylated DRD1 liposome and control mock liposome. Among 764 clones examined, 118 clones specifically bound to antigen DRD1 (more than 10-fold signal intensity was detected in DRD1 liposome compared with mock liposome) in ELISA and/or BiLIA (Fig. 2c, spots in area A–C). Interestingly, many clones with high ELISA responses (Fig. 2c, area B) showed low signal intensity in BiLIA, suggesting that each screening method recognizes different types of anti-DRD1 antibodies.

To clarify the differences between ELISA and BiLIA, 143 clones with different scores in both assays were selected and subjected to Western blotting (WB) and immunoprecipitation (IP) (Fig. 2d and Supplementary Table S2 online). WB detects binding between antibody and denatured linear antigen peptide immobilized on membrane, whereas native tertiary structure of antigen protein is kept in IP^28^. Almost all of the BiLIA-positive clones (56/57, 98%) were WB-positive/IP-positive (WB^+^/IP^+^), whose epitope was exists on the surface of native protein, or WB^–^/IP^+^, conformation-sensitive antibodies. It also should be noted that WB^–^/IP^+^ accounted for 47.5% of top 40 clones, and 7 of top 10 clones were WB^–^/IP^+^. In ELISA-positive clones, on the other hand, WB^+^/IP^+^ and WB^–^/IP^+^ accounted for 85% (79/92) of them. WB^–^/IP^+^ clones were 27.5% in top 40 clones, and only three WB^–^/IP^+^ were in the top 10. There were eight WB^+^/IP^–^ antibodies in 143 supernatants, whose epitope sequence is hidden inside of the native protein. No WB^+^/IP^–^ was BiLIA-positive, and only one antibody showed weak binding signal (7.3-fold DRD1/mock ratio). However, five out of eight WB^+^/IP^–^ were ELISA-positive (>10-fold ratio). These results implied the difference in antibody selection between these two methods, and BiLIA would preferably select conformation-sensitive antibody.

For a more detailed characterization, we selected 19 WB^+^/IP^+^ and 17 WB^–^/IP^+^ clones (Fig. 2d, arrowheads). Isotypes of the 36 mAbs were determined as follows: IgG1, 7; IgG2a, 11; IgG2b, 18 (Supplementary Table S2 online).

### Production of rabbit mAbs by ISAAC

In addition to mouse mAb, we also produced rabbit anti-DRD1 mAbs using immunospot array assay on a chip (ISAAC)^29^,^30^. Rabbit antibody has an advantage in its extremely high affinity and is expected to be a powerful tool for functional analysis and diagnosis. However, immunization of rabbit requires several mg of purified antigen proteins, which obstructs anti-membrane protein antibody production in rabbit. We noticed that bilayer-dialysis method was able to provide enough amount of GPCR antigen for immunizing rabbit. We prepared 5 mg of purified DRD1, and immunized a rabbit with it. After sacrifice, peripheral blood lymphocytes, spleen and bone marrow cells were collected and rabbit IgG^+^ cells were concentrated and spread on a microwell array chip. Anti-DRD1-specific antibody-secreting cells were identified using DRD1-liposome probe, which was prepared by using liposome containing 10% (w/w) biotin-CAP-DPPE, 5% (w/w) PEG1000-phosphoethanolamine, and 85% (w/w) asolectin. The variable regions of heavy chain and light chain were amplified by single-cell RT-PCR. Among 146 antibodies expressed in cultured cells, 10 clones specifically reacted with DRD1. After subtracting sequence redundancy of antibodies, we finally obtained six unique mAb clones for DRD1 (Supplementary Table S3 online). All the rabbit antibodies obtained in this study were applicable to ELISA, BiLIA, IP and WB, respectively.

### Characterization of anti-DRD1 mAbs

We evaluated affinity of the antibodies by using Scatchard plot with ELISA^30^,^31^, because the dissociation between some antibodies and antigen was too slow to determine kinetics with SPR. K_d_ values of mouse mAbs ranged from 10^–7^ M to 10^–10^ M (Fig. 2e). Half mouse mAbs (19/36) showed relatively low affinity with K_d_ ≈ 10^–8^ M, and the left showed higher affinity as much as 5.5 × 10^–11^ M. On the other hand, all of the rabbit anti-DRD1 antibodies showed high affinity with K_d_ values from 7.8 × 10^–10^ M to 8.4 × 10^–11^ M. Determined K_d_ values of mouse mAbs were not correlated with ELISA or BiLIA scores in primary screening (Supplementary Fig. S1 online).

We performed immunostaining using anti-DRD1 mAbs obtained in this study. Proximity ligation assay (PLA)^32^ successfully detected overexpressed DRD1-V5 fusion protein in HeLa cells with 25 out of examined 36 mouse mAbs and all of six rabbit mAbs (Fig. 2f-i, Supplementary Table S2 online and Supplementary Table S3 online). The localization of PLA signals detected by anti-DRD1 mAb staining was identical to positive control anti-V5 antibody staining. Among 11 immunostaining-negative clones, 10 mAbs were WB-negative (Supplementary Table S2 online). Although not all WB-negative mAbs failed to stain DRD1-expressed cells, its success rate (41.2%) was significantly lower than that of WB-positive clones (96%). DRD1 might be denatured in some extent when fixed by 4% paraformaldehyde, and cannot be recognized by conformation-sensitive mAbs.

### Epitope mapping

**Figure 3:**
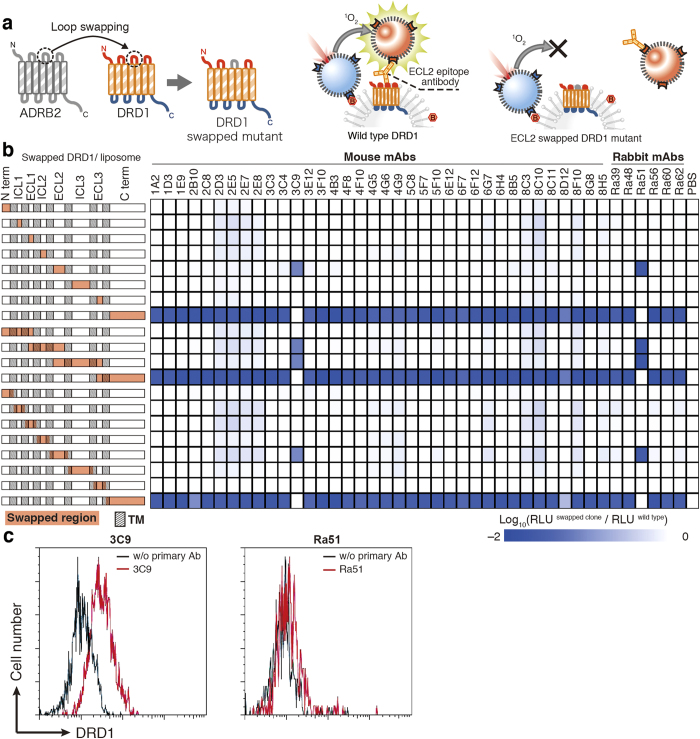
Epitope mapping using swapping DRD1 mutants. (**a**) A schematic diagram of epitope mapping using swapped mutant. An arbitrary part of DRD1 was swapped with homologous region of other GPCRs by PCR and In-Fusion assembly. Binding between mAb clones and swapped mutants was evaluated by BiLIA. (**b**) Binding of mAbs toward swapped mutants. The left of figure shows swapped region (red area) and transmembrane (shaded box) of each swapped clone. Intense blue in the right plots indicates that the mAb did not bind to the swapped mutant. (**c**) Staining profiles of DRD1-overexpressed cell with DRD1-specific antibodies. HeLa cells were transfected with pIRES2-AcGFP/DRD1, and stained with DRD1-specific antibodies 3C9 (left) or Ra51 (right) followed by Alexa Fluor 647-conjugated anti-mouse (left) or anti-rabbit (right) IgG-specific antibody, respectively. Stained cells were analyzed with a flow cytometer. Transfected cells were gated on the basis of GFP expression. Horizontal axis indicates the log intensities of fluorescence; vertical axis indicates cell number. Representative histograms in two independent experiments are shown.

To determine the epitope of anti-DRD1 mAbs, loop-swapped mutants of DRD1 were prepared as below. The region harboring loop or terminus region of DRD1 was swapped with the homologous fragment of ADRB2 or HRH2 using PCR and In-Fusion assembly (Fig. 3a). Constructed DRD1 mutants were synthesized on biotinylated liposome using wheat cell-free system with bilayer method, and interaction between biotinylated DRD1 liposome and mAbs was analyzed by BiLIA. When the region containing epitope was swapped, BiLIA signal decreased. Among the mAbs examined, 40 mAbs recognized C-terminus region of DRD1 (Fig. 3b). It was further confirmed by using soluble recombinant proteins fused with epitope fragments (N-biotin-*Staphylococcus aureus* SrtA-epitope fragment-C, Supplementary Fig. S2 online)^33^. BiLIA demonstrated that all the mAbs bound to the fusion proteins. Among 40 C-terminus recognizing mAbs, 39 mAbs bound to C terminus 37 amino-acid fragments (411–447), and only rabbit mAb clone Ra48 bound to fragment 374–413. Only two notable exceptions, mouse mAb clone 3C9 and rabbit Ra51, recognized DRD1 extra cellular loop 2 (ECL2). We further analyzed whether these antibodies bind to DRD1 on the surface of living cells using flow cytometry. DRD1 was overexpressed in HeLa cells, treated with each antibody, and subjected to flow cytometer without permeabilization and fixation. The histogram of clone 3C9-treated cells shifted to the right, indicating that it bound to HeLa cells expressing DRD1 (Fig. 3c). On the other hand, the histogram of Ra51 did not show shift obviously.

### Specificity of anti-DRD1 mAbs

**Figure 4:**
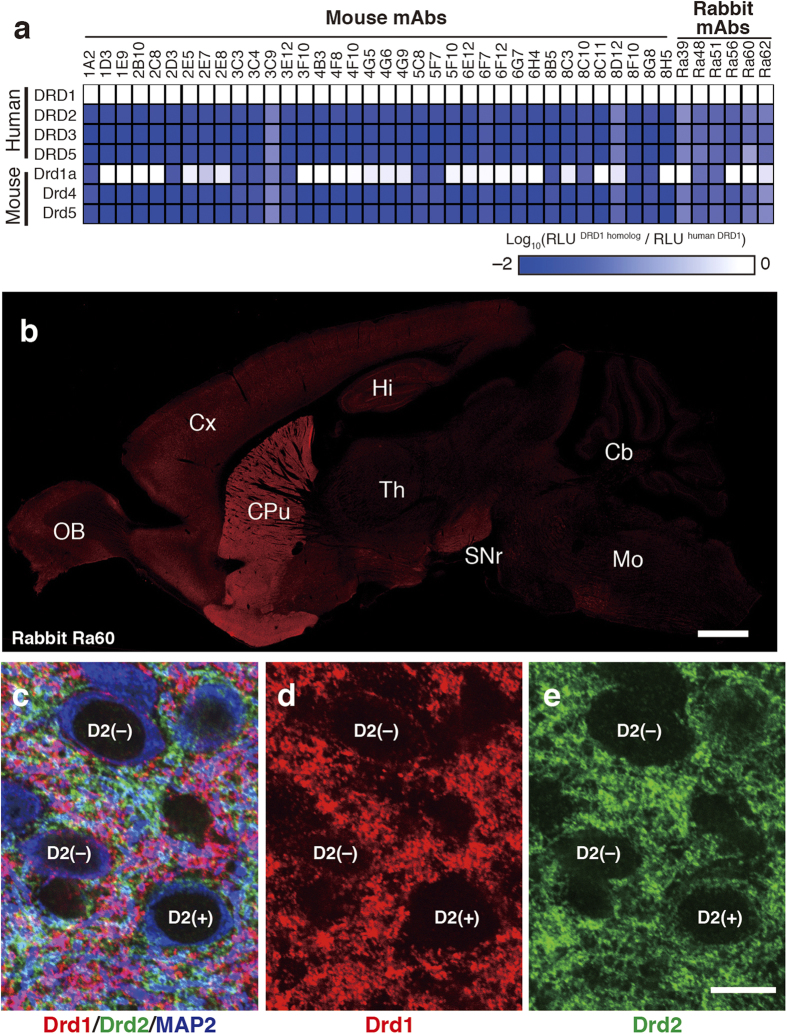
Subtype specificity of anti-DRD1 mAbs. (**a**) Cross reactivity against human DRD1 homologs determined by BiLIA. White indicates that the mAb bound to the homolog. (**b**) DRD1 distribution in mouse brain detected by rabbit mAb obtained in this study. The sagittal plane section was stained by rabbit mAb clone Ra60. Scale bar: 1mm. (**c**–**e**) Distribution of dopamine receptors in mouse striatum. Red, rabbit anti-DRD1 mAb clone Ra60; green, guinea pig anti-DRD2 polyclonal^36^; blue, goat anti-MAP2 polyclonal antibody^37^. Neurons with Drd2-negative and Drd2-positive perikarya are labeled with D2(–) or D2(+), respectively. Scale bar: 10 μm.

Cross-reactivity of obtained mAbs was examined using three human DRD1 paralogs and three mouse DRD1 homologs. We synthesized these six DRD1 homologs on biotinylated liposome using bilayer method, and detected bindings between DRD1 homologs and mAbs using BiLIA. Twenty-four mouse mAbs and three rabbit mAbs did not bind any DRD1 homologs (Fig. 4a). Other 12 mouse mAbs and three rabbit mAbs bound to mouse Drd1a, but did not cross-reacted with other homologs. Epitope of these mAbs cross-reacted with mouse Drd1a was C-terminus (411–447) without exception. The two ECL2-recognizing mAbs (mouse 3C9 and rabbit Ra51) only recognized human DRD1.

Specimen of the mouse brain was stained by rabbit mAb clone Ra60, which cross-reacted with mouse Drd1a (Fig. 4b). Intense labeling signal for DRD1 was detected in the striatum. Weaker signal was also observed in the substantia nigra, olfactory bulb and cortex. This distribution pattern is consistent with that in previous studies by immunohistochemistry (IHC) or *in situ* hybridization^34^,^35^. In the striatum, rabbit anti-DRD1 mAb clone Ra60 was well overlapped with guinea pig anti-Drd1 polyclonal antibody (Supplementary Fig. S3 online), which specifically detects Drd1 in IHC^36^. Furthermore, when mouse striatum section was triple immunostained by rabbit anti-DRD1 mAb clone Ra60, guinea pig anti-Drd2 polyclonal antibody^36^ and goat anti-microtubule-associated protein 2 (MAP2) polyclonal antibody^37^, complementary labeling pattern of Drd1 and Drd2 signals were observed in neurophils (Fig. 4c–e). Perikaryal labeling of Drd1 was not observed in Drd2^+^/MAP2^+^ cells (0%, n = 94), whereas weak Drd1 labeling signal was detected in 90.9% of Drd2^–^/MAP2^+^ perikarya (n = 88) (Supplementary Fig. S4 online). These results strongly demonstrate the high specificity of the rabbit anti-DRD1 mAb produced in this study.

### Antibody production of additional three GPCR targets

**Figure 5:**
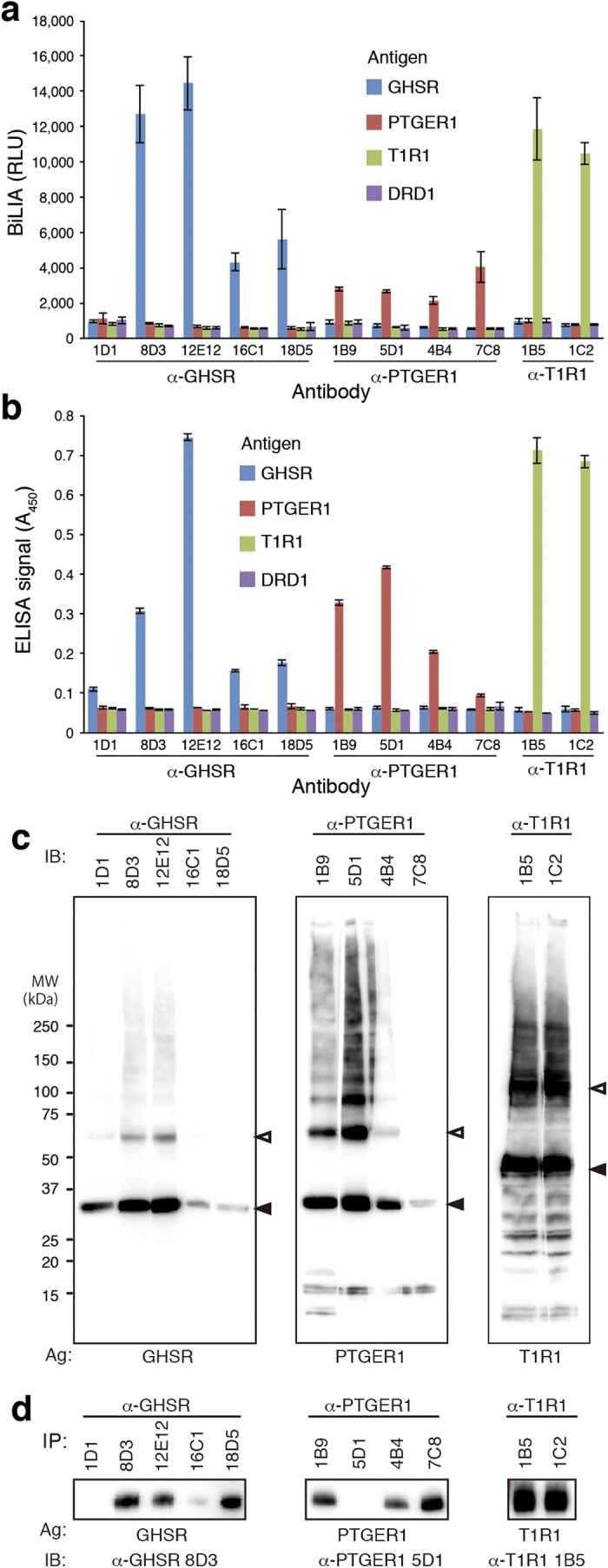
mAbs against three GPCRs. (**a**) BiLIA. (**b**) ELISA. (**c**) Western blotting. Cell-free synthesized GPCR antigens were subjected to SDS-PAGE and blotted on a PVDF membrane. The blotted membrane was cut into rectangles to separate lanes, and each piece was treated with the corresponding mAb, respectively. The immunoreactive signals were visualized by anti-mouse IgG-HRP and ECL reagent. Filled and blank arrowheads indicate monomer and dimer band of GPCR antigen, respectively. (**d**) Immunoprecipitation. GPCR/liposome complex was incubated with antibody-captured magnetic beads. Immunoprecipitated GPCR was visualized by Western blotting.

In addition to DRD1, we also produced mAbs against another three targets, GHSR (class A), PTGER1 (class A) and T1R1 (class C), using cell-free synthesized antigen. Productivity of GHSR, PTGER1 and T1R1 in bilayer-dialysis method was approximately 10 mg/mL, 5 mg/mL, and 5 mg/mL wheat germ extract, respectively. One mg of each receptor was synthesized using bilayer-dialysis method, and immunized to mice. After antibody screening by BiLIA and ELISA (Fig. 5a,b), four mAbs against GHSR1a, four mAbs against PTGER1, and two anti-T1R1 mAbs were obtained. Results of WB and IP are shown in Fig. 5c and 5d, respectively. Among 11 mAbs, two antibodies (WB^–^/IP^+^) were found to be conformation-sensitive, anti-GHSR 18D5 and anti-PTGER1 7C8. Both antibodies showed high reactivity in BiLIA compared with ELISA. On the other hand, anti-GHSR 1D1 and anti-PTGER1 5D1 were WB^+^/IP^–^, suggesting that they recognize the epitope exposed by denaturation. Anti-GHSR 1D1 did not respond in BiLIA, and anti-PTGER1 5D1 showed relatively weak BiLIA signal compared with ELISA. These results also indicate that BiLIA would preferably select conformation-sensitive antibody, and suggest that the approach in this study is highly useful for the production of mAbs against GPCR.

## Discussion

Purified full-length membrane protein with correct tertiary structure is regarded as ideal antigen in anti-membrane protein mAb production. However, cellular overexpression system has some technical limitations such as low expression and aggregation^1^,^2^,^4^. Our bilayer-dialysis method has potential to overcome these problems with high yield and success rate. In this study, we demonstrated that 25 GPCRs have been prepared successfully using the bilayer-dialysis method, and dozens of anti-GPCR antibodies against four GPCRs were produced and selected using cell-free synthesized GPCR as antigen. Especially, we obtained over 40 anti-DRD1 mAbs binding to native DRD1 with high affinity and specificity, and they can be applicable to a wide range of studies including IHC. Our results show that cell-free synthesized GPCRs retain the sufficient quality and quantity for immunization. One to several mg of partially purified GPCRs can be prepared with 1 mL of WEPRO7240 wheat germ extract (Fig. 1e), which is enough to prepare large amount of GPCR antigens cost-effectively even for immunizing big animals.

Cell-free synthesis exhibits superior productivity and success rate compared with other expression systems; however, cell-free synthesized GPCR antigens still remain some problems to be solved. Topology of receptor on the liposome is one of them. Cell-free synthesized DRD1 was bidirectional because both ECL2-epitope mAbs and C-terminus-epitope mAbs bound to biotinylated DRD1 liposome equally in BiLIA (Fig. 3b). We assumed that cell-free synthesized membrane proteins physicochemically penetrate lipid layer via its hydrophobic domain because SEC translocon system is absent in either wheat cell-free system or liposome, and thus the direction of the molecule was random. To acquire druggable antibodies that bind to the extracellular loop of the receptor and control its biological function, extracellular side of GPCR antigen is required to point towards the outside of the liposome. Further technological developments are needed to control topology of GPCR on liposome freely as desired. Glycosylation is an important difference between cell-free synthesized GPCR and GPCR expressed in cellular system. Because cell-free system does not contain ER or Golgi apparatus, the product is not glycosylated in principal. Lack of glycosylation may affect stability or activity of cell-free synthesized protein. DRD1 also has N-linked glycosylation residue at the N terminus, and cell-free synthesized DRD1 might have limited relative activity due to lack of glycosylation. On the other hand, cell-free synthesized glycosylation-free antigen can be advantageous when antibody against glycosylation site should be avoided.

In addition to antigen preparation method, we also developed a new assay method, BiLIA, to detect interaction between antibody and GPCR antigen on the liposome using biotinylated proteoliposome and AlphaScreen (Fig. 2a). We prepared biotinylated GPCR liposome using bilayer method (Fig. 1a), because it can be conducted on 96-well plate, and suitable for preparation of a large number of samples at the same time. Especially it is advantageous for epitope mapping (Fig. 3) or isotype specificity analysis (Fig. 4a), which requires mutant antigens or family GPCRs. As our study show, BiLIA preferentially detects conformation-sensitive antibodies (WB^–^/IP^+^) and tends to ignore WB^+^/IP^–^ antibodies that can not bind to native protein. On the contrary, ELISA recognizes several WB^+^/IP^–^ antibodies as positive. We think the above difference may be due to the environment of antigen molecules. BiLIA is bead-based in-solution assay, and thus antigen membrane protein is neither immobilized on the solid state nor exposed to air, which theoretically contributes to preventing antigen membrane proteins from denaturation.

We successfully produced monoclonal antibodies using cell-free synthesized proteoliposome antigen in this study (Fig. 3 and Fig. 5). The number of the examples in this study is not big enough to compare the performance of the present method with other conventional methods, such as DNA immunization, whole cell immunization, virus-like particle and/or reconstituted proteoliposome. However, the present method has several advantages; one feature is high success rate of GPCR antigen production. Although the 25 GPCRs examined in this study are only a small portion among 800 GPCRs existing in human genome, there is no doubt that our cell-free based approaches have the potential to be applied to most GPCRs analyses considering its superior success rate. It also should note that these 25 GPCRs have wild-type amino acid sequence without any purification tag, amino acid substitution and T4L/BRIL fusion (Supplementary Table S1 online), which avoids risks of undesired epitopes derived from mutations of GPCR antigens. When a target GPCR is difficult to be expressed in cellular system, cell-free approach should be considered. Furthermore, we previously reported that a variety of membrane proteins with multiple transmembrane domains are successfully synthesized using cell-free system^15^. Application of these technologies should be not only, of course, limited to GPCRs, but also generally applicable to the examinations of other membrane proteins. We believe that the presented methods should be able to promote the production and functional analysis of antibodies against wide variety of membrane proteins.

## Methods

### Wheat Cell-free synthesis and purification of GPCR

Open reading frame of human GPCR was amplified by PCR using full-length cDNA clones from Mammalian Gene Collection^38^ or Flexi ORF clone (Promega)^39^ as templates (Supplementary Table S1 online). Amplified PCR product was subcloned into pEU-E01 vector^24^ using Gateway system (Life Technologies). *In vitro* transcription was performed by using SP6 polymerase. Pre-treatment of asolectin was conducted as previously reported^15^. We utilized two *in vitro* translation methods according to the assay purposes and amount (Fig. 1). Bilayer method was conducted as described before^15^,^24^. Twenty-five μL of reaction mixture containing 10 μL of WEPRO7240 wheat germ extract (Cell-Free Sciences), 10 μL of mRNA, 40 μg/mL creatine kinase, and 10 mg/mL asolectin liposome was overlaid with 125 μL of SUB-AMIX SGC solution (Cell-Free Sciences) in a flat-bottom 96-well titer-plate, and incubated at 15 °C for 24 h. For large-scale membrane protein preparation, bilayer-dialysis method was used and conducted as follows. Five-hundred μL of reaction mixture (125 μL of WEPRO 7240 wheat germ extract, 125 μL of mRNA, 40 μg/mL creatine kinase, and 10 mg/mL asolectin liposome) was overlaid with 2 mL of SUB-AMIX SGC solution (Cell-Free Sciences) in a 10-K MWCO Slide-A-Lyzer dialysis device (Thermoscientific), then the cup was immersed in 3.5 mL dialysis solution (SUB-AMIX SGC). The reaction was carried out at 15 °C for 48 h. The dialysis solution was replaced after 24 h.

Purification of cell-free synthesized GPCR was conducted as follows. GPCR/liposome complex was collected by centrifugation at 20,000 × g for 10 min at 4 °C, and resultant pellet was washed with sterilized PBS for 3 times. For biochemical assay and immunization, the pellet was finally re-suspended in a small amount of sterilized PBS. For further purification, GPCR was solubilized and purified by SEC^6^,^19^. A partially purified GPCR/liposome complex was collected by centrifugation as described above, and the pellet was suspended in 300 μL of solubilization solution (150 mM sodium bicarbonate, 750 mM NaCl, 4% (w/v) DDM, 10% glycerol and 1 mM dithiothreitol). The suspension was then sonicated using SONIFIER model 450 Advanced (BRANSON) at 20 °C for 15 min, then centrifuged at 14,000 × g and 4 °C for 15 min. The supernatant containing solubilized GPCR was collected and was loaded onto a Superdex 200 10/300 24-mL SEC column (GE Healthcare) and eluted with SEC buffer containing 20 mM Hepes-NaOH (pH 7.0), 250 mM NaCl, 10% Glycerol, 1 mM DTT and 0.014% (w/v) 3 × CMC fos-choline-14 (Affymetrix). Gel Filtration Markers (Sigma, 6,500-66,000 Da) was used as Molecular weight standards. A_280_ of each fraction was measured by spectrophotometer (DU640, Beckman Coulter).

### Electron microscopy

DRD1-biotin/liposome complex or biotinylated liposome was subjected to electron microscopy using freeze-fracture replica labeling method, respectively^40^. The replica was labeled with anti-biotin antibody, followed by colloidal gold-conjugated secondary antibody. DRD1-biotin ligation site fusion protein was synthesized on liposomes and enzymatically biotinylated by BirA biotin ligase^23^. Biotinylated liposome was made from biotin-cap DPPE and asolectin mixture (1:99, w/w).

### Biacore assay

Specific binding between DRD1 and its ligands was also examined by Biacore X100 apparatus (GE healthcare). The running buffer was HBS-EP+, containing 10 mM Hepes-NaOH (pH 7.4), 150 mM NaCl, 0.05% (v/v) Tween 20, and 3 mM EDTA. The temperature of the flow cells was kept at 25 °C. Dopamine was immobilized on detection cells of CM5 sensor chip (GE healthcare) and histamine was immobilized on reference cells respectively, by using standard amine coupling chemistry. The immobilization level of dopamine and histamine is around 200 RU and 150 RU, respectively. Solubilized and SEC-purified DRD1 (wild-type or S198A/S199A mutant, from 74 nM to 4.7 μM) was injected into the flow cells at 30 μL/min for 120 s. Affinity was calculated by BIAEvaluation software.

### BiLIA

Interaction between antibodies and membrane proteins on the surface of biotinylated liposome was assayed by AlphaScreen. Biotin-cap DPPE (Avanti) and asolectin (Sigma) was solubilized in chloroform respectively, and mixed in a vial (1:99 w/w). Chloroform was evaporated by a nitrogen stream. Lipid film was dried completely under vacuum for more than 1 h. The lipid film was then hydrated by adding SUB-AMIX SGC solution (100 mg lipid/mL), and the biotinylated liposome was homogenized by sonication. DRD1/biotinylated liposome complex was synthesized with bilayer method using biotinylated liposome instead of asolectin liposome. One μL of DRD1/biotinylated liposome complex was mixed with 2 μL of culture supernatant of a hybridoma cell line in 100 mM Tris-HCl (pH 8.0), 100 mM NaCl, and 1 mg/mL BSA, 0.1 μL of AlphaScreen streptavidin coated donor beads and 0.1 μL of AlphaScreen protein A conjugated acceptor beads (PerkinElmer) in 25 μL reaction mixture. After 1 h incubation at 25 °C in dark chamber, AlphaScreen chemiluminescence signals were detected by Envision reader (PerkinElmer).

### ELISA

DRD1 liposome was synthesized with bilayer method in the presence of asolectin liposome. As to the negative control, we also prepared mock translation reaction mixture without mRNA. Crude liposome sample was diluted 20 times, and 30 μL of the diluted liposome was injected in flat bottom clear 384-well plate and incubated overnight at 4 °C. After blocking with 50 μL of 5% skim milk in PBS with 0.2% tween, the plate was treated with culture media of hybridoma cells diluted by 5% skim milk in PBS. Antibodies bound to the plate were detected by anti-mouse IgG antibody-horseradish peroxidase (HRP) conjugates (Leinco) with *o*-phenylenediamine treatment.

### Preparation of anti-DRD1 mouse mAbs

Mouse monoclonal antibodies against DRD1 were developed by using conventional hybridoma technology from Abnova Corporation. We immunized mice subcutaneously with partially purified DRD1 liposome mixed with complete Freund’s adjuvant. Two, four, seven weeks after primary immunization, we boosted the mice with DRD1 liposome in incomplete Freund’s adjuvant. Finally the mice received an intraperitoneal injection of DRD1 liposome at 10 weeks after primary immunization. Three days after final boost, the mice were sacrificed and spleen cells were fused with SP2 myeloma cell line. Hybridoma cells were cloned by limiting dilution.

### Binding mode analysis of anti-DRD1 mAbs

Binding against denatured antigen was detected by WB and binding to native antigen was evaluated by IP. In WB, crude DRD1 liposome was applied to SDS-PAGE and blotted to PVDF membrane using iBlot transfer system (Life Technologies). The blotted membrane was cut into strips, treated with 5% milk in tris-buffered saline containing 0.01% tween20 (TBST). Each piece of sprit was probed with each hybridoma supernatant diluted 50-fold by 5% milk, followed by HRP-conjugated anti-mouse IgG antibody (GE healthcare) treatment. Antibody on the membrane strip was visualized by Immunostar reagent (Wako).

IP was conducted using dynabeads protein G kit (Life Technologies) with radioisotope-labeled DRD1. Ten μL of hybridoma culture medium or purified antibody was incubated with protein G magnetic beads for 10 min at room temperature. Antibody capturing beads were then mixed with radioisotope-labeled DRD1 that is synthesized using wheat cell-free system in the presence of [^14^C]-Leu (PerkinElmer) and liposome. After a 10-min incubation, the beads were washed 3 times by PBS, and suspended in SDS-PAGE sample buffer, incubated at 65 °C for 15 min. Supernatant was applied to SDS-PAGE, and autoradiography image was acquired by using Typhoon FLA 7000 (GE healthcare).

### Preparation of anti-DRD1 rabbit mAbs by ISAAC

Rabbit experiments were approved by the Committee on Animal Experiments at the University of Toyama. We immunized 12- to 13-week-old New Zealand White rabbits (Sankyo Lab) subcutaneously with 0.5 mg partially purified DRD1/asolectin liposome complex in complete Freund’s adjuvant. Two, four, six and eight weeks after primary immunization, we boosted the rabbit subcutaneously with 1 mg DRD1 liposome in incomplete Freund’s adjuvant. One week after the final boost, we collected cells from peripheral blood lymphocytes, spleen and bone marrow, and isolated rabbit IgG^+^ cells with rabbit IgG-specific antibody-conjugated microbeads (Miltenyi Biotec) using an autoMACS Pro separator (Miltenyi Biotec) according to the manufacturer’s instructions. ISAAC was conducted as described in our previous report with slight modifications^28^. Asolectin liposome containing 10% (w/w) biotin-CAP-DPPE and 5% (w/w) PEG1000-DPPE (Avanti) was prepared as described above, and DRD1 was synthesized on it. Surface of the ISAAC chip was coated with PBS-diluted rabbit IgG-specific antibody (MP Biomedicals) for 2 h at room temperature. After blocking with 0.01% Biolipidure (NOF Corporation, Japan) for 15 min at room temperature, cells were arrayed on the chip and incubated for 3 h to trap secreted IgG. The chip was then treated with DRD1/biotinylated liposome complex followed by treatment with Cy3-conjugated streptavidin (Sigma). After cells were stained with 1 mM Oregon Green (Molecular Probes), antibody-secreting cells were collected using micromanipulator (TransferMan NK2, Eppendorf) under a fluorescence microscope (BX51WI, Olympus). Isolation of mRNA, amplification and cloning of cDNA fragments of IgG by RT-PCR, and expression of rabbit IgG in CHO cells were performed as described previously^29^,^30^.

### Epitope mapping

Loop-swapped mutants of DRD1 were constructed as follows. pEU-E01-DRD1 plasmid was linearized by inverse PCR and arbitrary loop/terminus region was removed. The homologous loop/terminus region of ADRB2 or HRH2 was amplified by PCR. The PCR fragments were connected by using In-Fusion HD (Clontech). Wild-type and loop-swapped mutants were synthesized with bilayer method in the presence of biotinylated liposome. Antigen-antibody reaction was assayed by BiLIA as described above.

### Immunostaining of cultured cells

DRD1 ORF was subcloned into pcDNA3.2/V5 dest vector (Life Technologies) by using Gateway technology, and the resultant plasmid was transfected into HeLa cells. Transfected cells were fixed by 4% paraformaldehyde and permeabilized by 0.5% TritonX-100. *In situ* PLA was performed with Duolink *In Situ* kit (Olink bioscience) according to the manufacturer’s instructions. For mouse mAb detection, both anti-mouse PULS and anti-mouse Minus PLA probes were used. Rabbit mAb was detected by using anti-Rabbit PLUS and MINUS PLA probes.

### Determination of affinity

We determined antibody affinities (Kd) at antibody-antigen equilibrium in solution using ELISA and Scatchard plot analysis^30^,^31^. DRD1 epitope fragment (ECL2 or C-terminus region of DRD1) was fused with FLAG-GST tag respectively, and synthesized using wheat cell-free system. The fusion proteins were affinity-purified by using glutathione sepharose 4B (GE healthcare). Various concentrations (0.5, 1, 5 or 10 nM) of mAbs were incubated with 3 to 200 nM of DRD1 epitope fusion proteins overnight at 4 °C until equilibrium was established. We then measured the concentrations of free antibody in the mixture by ELISA as described above. Kd value was determined by Scatchard plot analysis using the concentration of free antibody that was unsaturated by antigen [Abf] and the concentration of free antigen that was not bound to antibody [Agf] in the mixture.

### Flow cytometry

DRD1 ORF was inserted into multiple cloning site of pIRES-AcGFP (Clontech). HeLa cells were transfected with pIRES2-AcGFP/DRD1. Transfected cells were incubated with 5 μg/mL of DRD1-specific mouse or rabbit monoclonal antibodies for 15 min at room temperature. The cells were then washed and stained with Alexa Fluor 647-conjugated anti-mouse or rabbit IgG specific antibody (Molecular Probes) for 15 min at room temperature. After being washed, the cells were analyzed with a FACSCanto flow cytometer (Becton Dickinson).

### Immunohistofluorescence

Mouse brain section was co-stained using antibodies against anti-DRD1 rabbit monoclonal antibody clone Ra60 (this study), anti-Drd1 guinea pig polyclonal antibody, anti-Drd2 guinea pig polyclonal antibody^36^, and anti-MAP2 goat polyclonal antibody^37^. C57BL6 mice were fixed by transcardial perfusion with 4% paraformaldehyde/0.1 M phosphate buffer (pH 7.2). Brains were removed from the skull, and cut with a microslicer (VT1000S, Leica Microsystems). All immunofluorescence incubations were conducted with the free-floating method at room temperature. Brain slices were sequentially subjected to the followings: 10% normal donkey serum, a mixture of primary antibodies, and a mixture of species-specific secondary antibodies conjugated with Alexa488, Cy3, or Alexa647 (Life Technologies, Jackson ImmunoResearch). To avoid the possibility for rabbit anti-DRD1 and guinea pig anti-Drd1 antibodies to compete with each other, we also adopted two-step staining method as follows. The first detection was performed using rabbit anti-DRD1 antibodies. After blocking with 10% normal rabbit serum, the second detection was performed with guinea pig anti-Drd1 and goat anti-MAP2 antibodies. PBS was used as diluting and washing buffer. Images were taken by a confocal laser-scanning microscope (FV1000; Olympus) equipped with helium–neon/argon laser and PlanApo (10×/0.40) and PlanApoN (60×/1.42, oil-immersion) objective lens (Olympus). To avoid cross-talk between multiple fluorophores, Alexa488, Cy3, and Alexa647 fluorescent signals were acquired sequentially using the 488, 543, and 633 nm excitation laser lines. Image analysis was performed using NIH ImageJ and Adobe photopshop CS3 (Adobe Systems).

## Additional Information

**How to cite this article**: Takeda, H. *et al.* Production of monoclonal antibodies against GPCR using cell-free synthesized GPCR antigen and biotinylated liposome-based interaction assay. *Sci. Rep.* **5**, 11333; doi: 10.1038/srep11333 (2015).

## Supplementary Material

Supplementary Information

Supplementary Information

## References

[b1] Bill R. M. *et al.* Overcoming barriers to membrane protein structure determination. Nat Biotechnol 29, 335–340 (2011). 21478852 10.1038/nbt.1833

[b2] Venkatakrishnan A. J. *et al.* Molecular signatures of G-protein-coupled receptors. Nature 494, 185–194 (2013). 23407534 10.1038/nature11896

[b3] Hopkins A. L. & Groom C. R. The druggable genome. Nat Rev Drug Discov 1, 727–730 (2002). 12209152 10.1038/nrd892

[b4] Hutchings C. J., Koglin M. & Marshall F. H. Therapeutic antibodies directed at G protein-coupled receptors. MAbs 2, 594–606 (2010). 20864805 10.4161/mabs.2.6.13420 PMC3011214

[b5] Rasmussen S. G. F. *et al.* Structure of a nanobody-stabilized active state of the β(2) adrenoceptor. Nature 469, 175–180 (2011). 21228869 10.1038/nature09648 PMC3058308

[b6] Hino T. *et al.* G-protein-coupled receptor inactivation by an allosteric inverse-agonist antibody. Nature 482, 237–240 (2012). 22286059 10.1038/nature10750 PMC3303121

[b7] Webb D. R., Handel T. M., Kretz-Rommel A. & Stevens R. C. Opportunities for functional selectivity in GPCR antibodies. Biochem Pharmacol 85, 147–152 (2013). 22975405 10.1016/j.bcp.2012.08.021 PMC3729224

[b8] Harding P. J. *et al.* Neurotensin receptor type 1: Escherichia coli expression, purification, characterization and biophysical study. Biochem Soc Trans 35, 760–763 (2007). 17635142 10.1042/BST0350760

[b9] Michalke K. *et al.* Mammalian G protein-coupled receptor expression in Escherichia coli: II. Refolding and biophysical characterization of mouse cannabinoid receptor 1 and human parathyroid hormone receptor 1. Anal Biochem 401, 74–80 (2010). 20175983 10.1016/j.ab.2010.02.017

[b10] Asada H. *et al.* Evaluation of the Pichia pastoris expression system for the production of GPCRs for structural analysis. Microb Cell Fact 10, 24 (2011). 21513509 10.1186/1475-2859-10-24 PMC3094209

[b11] Cherezov V. *et al.* High-resolution crystal structure of an engineered human beta2-adrenergic G protein-coupled receptor. Science 318, 1258–1265 (2007). 17962520 10.1126/science.1150577 PMC2583103

[b12] Hassaine G. *et al.* Semliki Forest virus vectors for overexpression of 101 G protein-coupled receptors in mammalian host cells. Protein Expr Purif 45, 343–351 (2006). 16055346 10.1016/j.pep.2005.06.007

[b13] Chelikani P., Reeves P. J., Rajbhandary U. L. & Khorana H. G. The synthesis and high-level expression of a beta2-adrenergic receptor gene in a tetracycline-inducible stable mammalian cell line. Protein Sci 15, 1433–1440 (2006). 16731977 10.1110/ps.062080006 PMC2265096

[b14] Fenalti G. *et al.* Molecular control of δ-opioid receptor signalling. Nature 506, 191–196 (2014). 24413399 10.1038/nature12944 PMC3931418

[b15] Nozawa A. *et al.* Production and partial purification of membrane proteins using a liposome-supplemented wheat cell-free translation system. BMC Biotechnol 11, 35 (2011). 21481249 10.1186/1472-6750-11-35 PMC3090341

[b16] Kalmbach R. *et al.* Functional cell-free synthesis of a seven helix membrane protein: *in situ* insertion of bacteriorhodopsin into liposomes. J Mol Biol 371, 639–648 (2007). 17586523 10.1016/j.jmb.2007.05.087

[b17] Jarecki B. W., Makino S., Beebe E. T., Fox B. G. & Chanda B. Function of Shaker potassium channels produced by cell-free translation upon injection into Xenopus oocytes. Sci Rep 3, 1040 (2013). 23301161 10.1038/srep01040 PMC3539143

[b18] Klammt C. *et al.* Cell-free production of G protein-coupled receptors for functional and structural studies. J Struct Biol 158, 482–493 (2007). 17350285 10.1016/j.jsb.2007.01.006

[b19] Kaiser L. *et al.* Efficient cell-free production of olfactory receptors: detergent optimization, structure, and ligand binding analyses. Proc Natl Acad Sci U S A 105, 15726–15731 (2008). 18840687 10.1073/pnas.0804766105 PMC2572932

[b20] Takemori N. *et al.* High-throughput synthesis of stable isotope-labeled transmembrane proteins for targeted transmembrane proteomics using a wheat germ cell-free protein synthesis system. Mol BioSyst 11, 361–365 (2015). 25431973 10.1039/c4mb00556b

[b21] Goren M. A., Nozawa A., Makino S.-i., Wrobel R. L. & Fox B. G. Cell-free translation of integral membrane proteins into unilamelar liposomes. Methods Enzymol. 463, 647–673 (2009). 19892197 10.1016/S0076-6879(09)63037-8 PMC5814320

[b22] Ullman E. F. *et al.* Luminescent oxygen channeling immunoassay: measurement of particle binding kinetics by chemiluminescence. Proc Natl Acad Sci USA 91, 5426–5430 (1994). 8202502 10.1073/pnas.91.12.5426 PMC44008

[b23] Sawasaki T. *et al.* Arabidopsis HY5 protein functions as a DNA-binding tag for purification and functional immobilization of proteins on agarose/DNA microplate. FEBS Lett 582, 221–228 (2008). 18082144 10.1016/j.febslet.2007.12.004 PMC7164004

[b24] Takai K., Sawasaki T. & Endo Y. Practical cell-free protein synthesis system using purified wheat embryos. Nat Protoc 5, 227–238 (2010). 20134421 10.1038/nprot.2009.207

[b25] Arimitsu E. *et al.* The ligand binding ability of dopamine D1 receptors synthesized using a wheat germ cell-free protein synthesis system with liposomes. Eur J Pharmacol 745, 117–122 (2014). 25446930 10.1016/j.ejphar.2014.10.011

[b26] Pollock N. J. *et al.* Serine mutations in transmembrane V of the dopamine D1 receptor affect ligand interactions and receptor activation. J Biol Chem 267, 17780–17786 (1992). 1355478

[b27] Matsuoka K., Komori H., Nose M., Endo Y. & Sawasaki T. Simple screening method for autoantigen proteins using the N-terminal biotinylated protein library produced by wheat cell-free synthesis. J Proteome Res 9, 4264–4273 (2010). 20575507 10.1021/pr9010553 PMC2917173

[b28] Mancia F. *et al.* Production and characterization of monoclonal antibodies sensitive to conformation in the 5HT2c serotonin receptor. Proc Natl Acad Sci U S A 104, 4303–4308 (2007). 17360519 10.1073/pnas.0700301104 PMC1838597

[b29] Jin A. *et al.* A rapid and efficient single-cell manipulation method for screening antigen-specific antibody-secreting cells from human peripheral blood. Nat Med 15, 1088–1092 (2009). 19684583 10.1038/nm.1966

[b30] Ozawa T. *et al.* A novel rabbit immunospot array assay on a chip allows for the rapid generation of rabbit monoclonal antibodies with high affinity. PLoS ONE 7, e52383 (2012). 23300658 10.1371/journal.pone.0052383 PMC3530603

[b31] Friguet B., Chaffotte A. F., Djavadi-Ohaniance L. & Goldberg M. E. Measurements of the true affinity constant in solution of antigen-antibody complexes by enzyme-linked immunosorbent assay. J Immunol Methods 77, 305–319 (1985). 3981007 10.1016/0022-1759(85)90044-4

[b32] Söderberg O. *et al.* Direct observation of individual endogenous protein complexes *in situ* by proximity ligation. Nat Methods 3, 995–1000 (2006). 17072308 10.1038/nmeth947

[b33] Matsunaga S., Matsuoka K., Shimizu K., Endo Y. & Sawasaki T. Biotinylated-sortase self-cleavage purification (BISOP) method for cell-free produced proteins. BMC Biotechnol 10, 42 (2010). 20525307 10.1186/1472-6750-10-42 PMC2901362

[b34] Levey A. I. *et al.* Localization of D1 and D2 dopamine receptors in brain with subtype-specific antibodies. Proc Natl Acad Sci USA 90, 8861–8865 (1993). 8415621 10.1073/pnas.90.19.8861 PMC47460

[b35] Lein E. S. *et al.* Genome-wide atlas of gene expression in the adult mouse brain. Nature 445, 168 (2007). 17151600 10.1038/nature05453

[b36] Narushima M., Uchigashima M., Hashimoto K., Watanabe M. & Kano M. Depolarization-induced suppression of inhibition mediated by endocannabinoids at synapses from fast-spiking interneurons to medium spiny neurons in the striatum. Eur J Neurosci 24, 2246–2252 (2006). 17042791 10.1111/j.1460-9568.2006.05119.x

[b37] Miura E. *et al.* Expression and distribution of JNK/SAPK-associated scaffold protein JSAP1 in developing and adult mouse brain. J. Neurochem. 97, 1431–1446 (2006). 16606357 10.1111/j.1471-4159.2006.03835.x

[b38] Strausberg R. L. *et al.* Generation and initial analysis of more than 15,000 full-length human and mouse cDNA sequences. Proc Natl Acad Sci USA 99, 16899–16903 (2002). 12477932 10.1073/pnas.242603899 PMC139241

[b39] Nagase T. *et al.* Exploration of human ORFeome: high-throughput preparation of ORF clones and efficient characterization of their protein products. DNA Res. 15, 137–149 (2008). 18316326 10.1093/dnares/dsn004 PMC2650635

[b40] Fujita A., Cheng J. & Fujimoto T. Quantitative electron microscopy for the nanoscale analysis of membrane lipid distribution. Nat Protoc 5, 661–669 (2010). 20360761 10.1038/nprot.2010.20

