# Supplementary material for: Production of monoclonal antibodies against GPCR using cell-free synthesized GPCR antigen and biotinylated liposome-based interaction assay
Source: Sci Rep. 2015 Jun 10;5:11333. doi: 10.1038/srep11333 (PMC4462149; doi:10.1038/srep11333)
Supplement: Supplementary Information [file srep11333-s1.pdf]

SUPPLEMENTARY INFORMATION

Production of monoclonal antibodies against GPCR using cell-free synthesized GPCR antigen and biotinylated liposome-based interaction assay

Hiroyuki Takeda, Tomio Ogasawara, Tatsuhiko Ozawa, Atsushi Muraguchi, Pei-Ju Jih, Ryo Morishita, Motokazu Uchigashima, Masahiko Watanabe, Toyoshi Fujimoto, Takahiro Iwasaki, Yaeta Endo, Tatsuya Sawasaki

Figure caption

Supplementary Figure S1 | Affinity of antibodies was not correlated with the scores in primary screening. Each plot indicates mouse mAb clone. Detailed data is shown in Supplementary Table 2 online.

Supplementary Figure S2 | Confirmation of epitope by using fusion protein. (a,b) Scheme of antibody-epitope fragment binding assay. ECL2 or C terminus fragments of DRD1 was fused with N-terminus biotinylated soluble protein (*Staphylococcus aureus* SrtA) and synthesized using cell-free system. Binding between biotinylated fusion proteins and antibodies are assayed by AlphaScreen. (a) antibody binds to epitope fusion protein and luminescence signal is generated. (b) when antibody fails to bind to epitope, AlphaScreen Acceptor bead does not generate luminescence signal. (c) Result of binding assay between antibodies and epitope fragments. Intense red color indicates the binding between the mAb and the epitope fragment.

Supplementary Figure S3 | Comparison of anti-DRD1 antibodies in Immunohistochemistry of the mouse striatum. Red, rabbit anti-DRD1 mAb clone Ra60 (this study); green, guinea pig anti-Drd1 polyclonal antibody[1]; blue, goat anti-MAP2 polyclonal antibody[2]. MAP2 is a marker for perikarya. Drd1-positive and Drd1-negative cells were indicated as D1(+) and D1(-), respectively. Scale bar: 10 µm.

Supplementary Figure S4 | The high-power fields of the triple-immunostaining IHC in Fig. 5c–e. Red, rabbit anti-DRD1 mAb clone Ra60; green, guinea pig anti-DRD2 polyclonal antibody; blue, goat anti-MAP2 antibody. Neurons with Drd2-negative and Drd2-positive perikarya are labeled with D2(-) or D2(+), respectively. Scale bar: 5 µm.

References

1. Narushima M, Uchigashima M, Hashimoto K, Watanabe M, Kano M. Depolarization-induced suppression of inhibition mediated by endocannabinoids at synapses from fast-spiking interneurons to medium spiny neurons in the striatum. *The European journal of neuroscience* **24**, 2246-2252 (2006).

2. Miura E*, et al.* Expression and distribution of JNK/SAPK-associated scaffold protein JSAP1 in developing and adult mouse brain. *Journal of neurochemistry* **97**, 1431-1446 (2006).

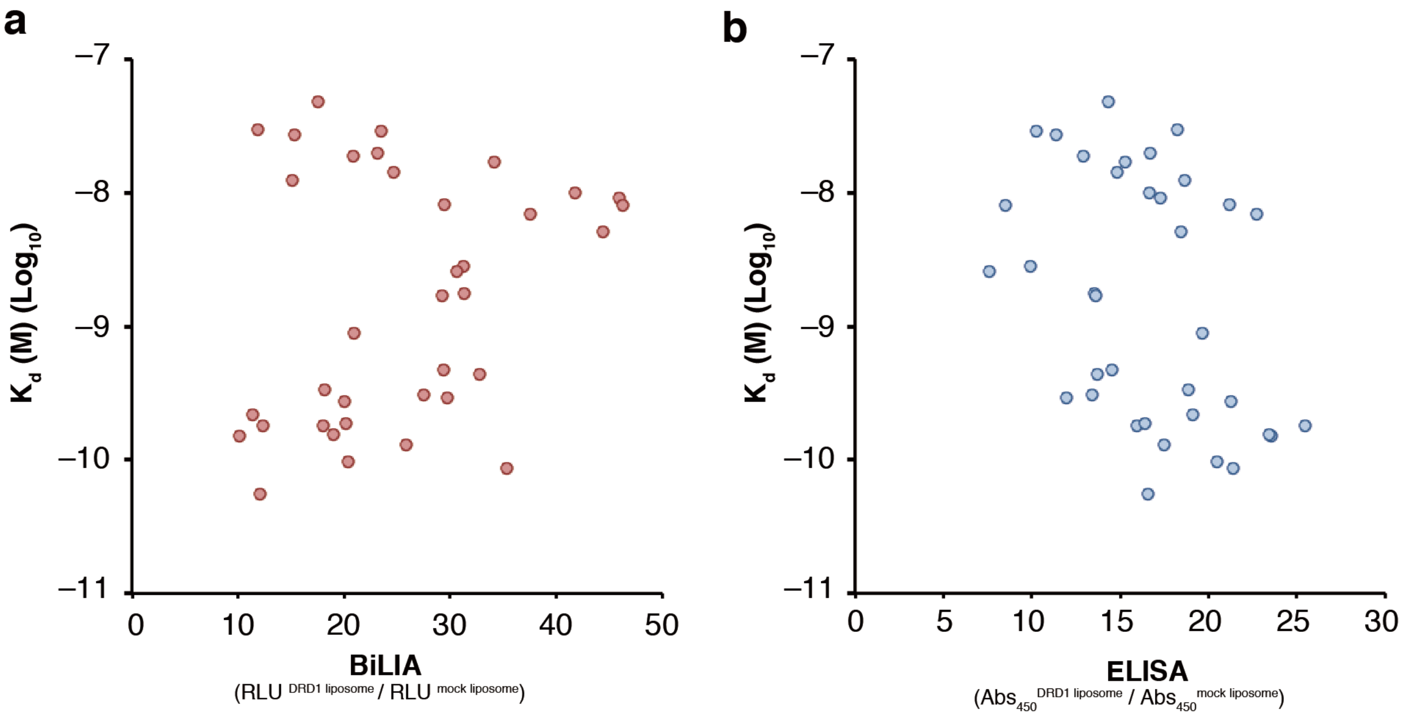

Supplementary Figure S1

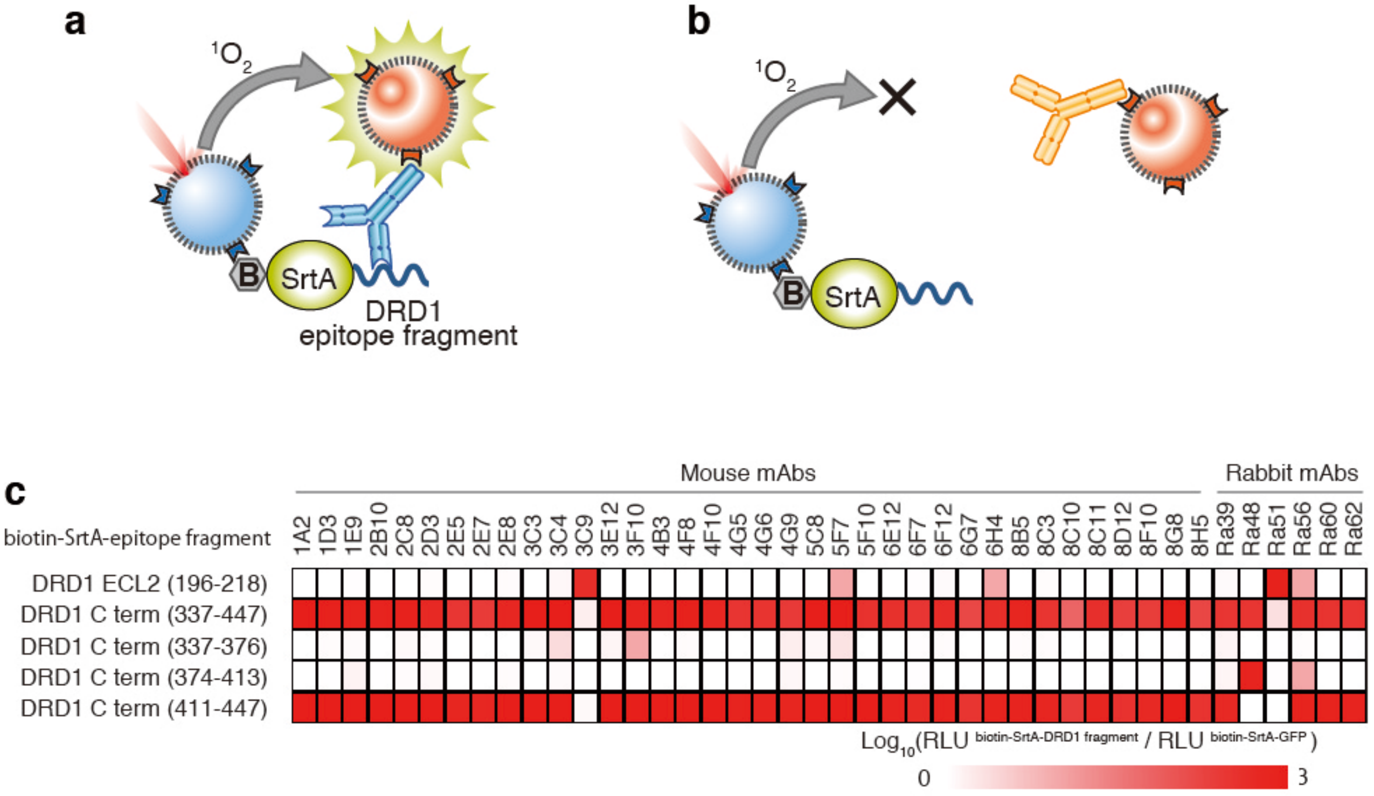

Supplementary Figure S2

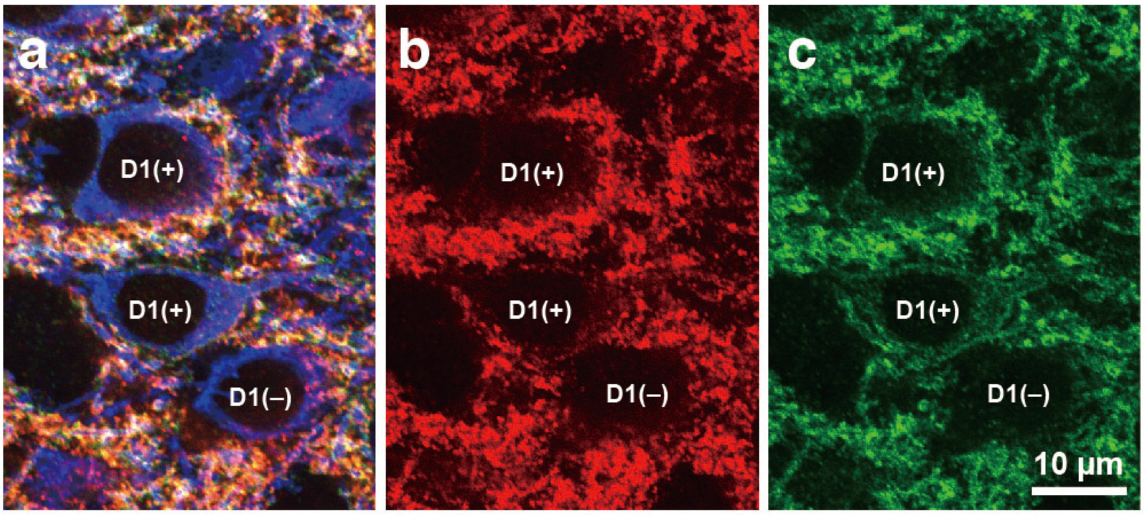

Drd1 (rabbit) /
Drd1 (guinea pig) / MAP2

Drd1 (rabbit) / MAP2

Drd1 (guinea pig) / MAP2

Supplementary Figure S3

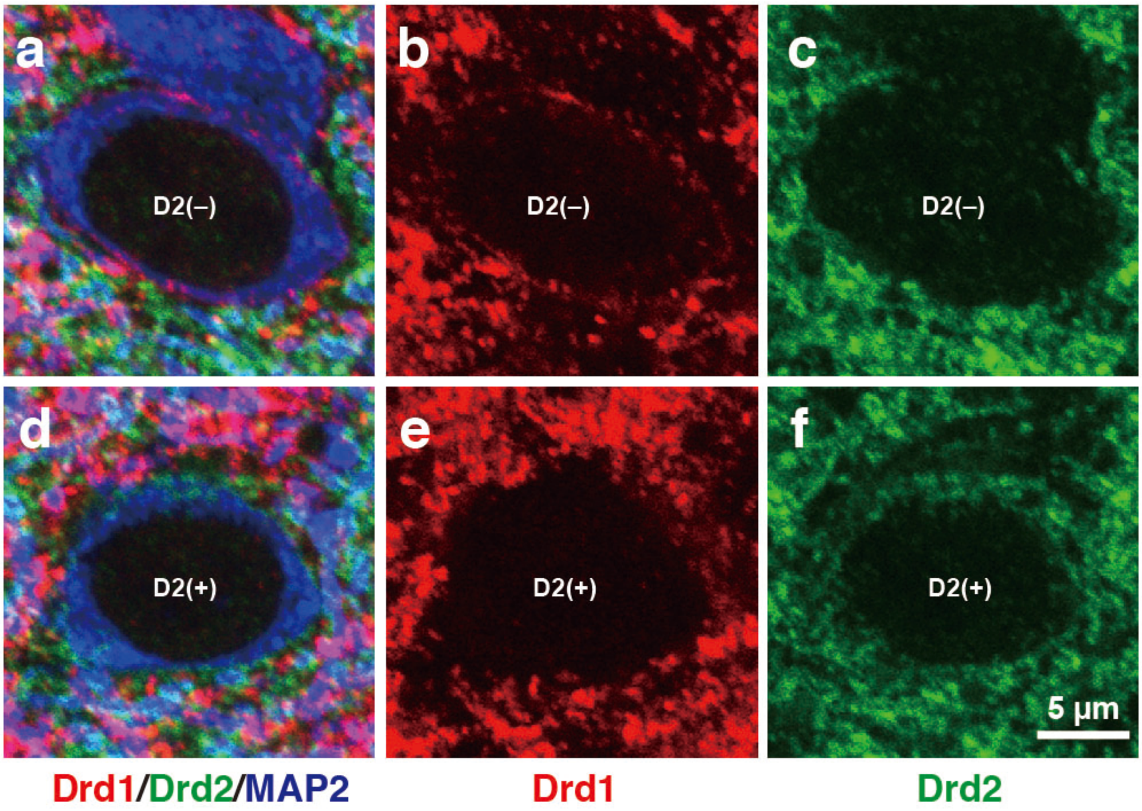

Supplementary Figure S4

Supplementary Table S1. 25 GPCRs synthesized in this study.

Symbol	GPCR class	Amino acid sequence	MW (kDa)	Resource[§]	Accession	Productivity in bilayer-dialysis method (mg/ mL wheat germ extract)
ADORA2A	Class A	MPIMGSSVYITVELAIAVLAILGNVLVCWAVWLNSNLQNVTNYFVVSLAAADIAVGVLAIPFAITISTGFCA<br>ACHGCLFIACFVLVLTQSSIFSLLAIAIDRYIAIRIPLRYNGLVTGTRAKGIIAICWVLSFAIGLTPMLGWNN<br>CGQPKEGKNHSQGCGEGQVACLFEDVVPMNYMVYFNFFACVLVPLLLMLGVYLRIFLAARRQLKQM<br>ESQPLPGERARSTLQKEVHAAKSLAIIVGLFALCWLPLHIINCFTFFCPDCSHAPLWLMYLAIVLSHTNS<br>VVNPFIYAYRIREFRQTFRKIIRSHVLRQQEPFKAAGTSARVLAAHGSDGEQVSLRLNGHPPGVWANG<br>SAPHPERRPNGYALGLVSGGSAQESQGNTGLPDVELLSHELKGVCPEPPGLDDPLAQDGAGVS	44.7	MGC clone	BC013780	4.0
ADRB2	Class A	MGQPGNGSAFLLAPNGSHAPDHDVTQERDEVWVVGMGIVMSLIVLAIVFGNVLVITAIAKFERLQTVT<br>NYFITSLACADLVMGLAVVPFGAAHILMKMWTFGNFWCEFWTSIDVLCVTASIETLCVIAVDRYFAITSP<br>FKYQSLLTKNKARVIILMVWIVSGLTSFLPIQMHWYRATHQEAINCYANETCCDFFTNQAYAIASSIVSFY<br>VPLVIMVFVYSRVFQEAKRQLQKIDKSEGRFHVQNLSQVEQDGRTGHGLRRSSKFCLKEHKALKTLGI<br>IMGTFTLCWLPFFIVNIVHVIQDNLIRKEVYILLNWIGYVNSGFNPLIYCRSPDFRIAFQELLCLRRSSLKA<br>YGNGYSSNGNTGEQSGYHVEQEKENKLLCEDLPGTEDFVGHQGTVPSDNIDSQGRNCSTNDSLL	46.5	MGC clone	BC073856	3.1
AGTR1	Class A	MILNSSTEDGIKRIQDDCPKAGRHNYIFVMIPTLYSIIFVVGIFGNSLVVIVIYFYMKLKTVASVFLLNLALA<br>DLCFLLTLPLWAVYTAMEYRWPFGNYLCKIASASVSFNLYASVFLLTCLSIDRYLAIVHPMKSRLRRTML<br>VAKVTCIIIWLLAGLASLPAIIHRNVFFIENTNITVCAFHYESQNSTLPIGLGLTKNILGFLFPFLIILTSYTLIW<br>KAVKKAYEIQKNKPRNDDIFKIIMAIVLFFFFSWIPHQIFTFLDVLIQLGIIRDCRIADIVDTAMPITICIAYFN<br>NCLNPLFYGFLGKKFKRYFLQLLKYIPPKAKSHSNLSTKMSTLSYRHSDNVSSSTKKPAPCFEVE	41.1	MGC clone	BC022447	5.1
CHRM2	Class A	MNNSTNSSNNSLALTSPYKTFEVVFIVLVAGSLSLVTIIGNILVMVSIKVNRHLQTVNNYFLFSLACADLII<br>GVFSMNLYTLYTVIGYWPLGPVVCDLWLALDYVVSNASVMNLLIISFDRYFCVTKPLTYPVKRTTKMAG<br>MMIAAAWVLSFILWAPAILFWQFIVGVRTVEDGECYIQFFSNAAVTFGTAIAAFYLPVIIMTVLYWHISRA<br>SKSRIKKDKKEPVANQDPVSPSLVQGRIVKPNNNNMPSSDDGLEHNKIQNGKAPRDPVTENCVQGEE<br>KESSNDSTSVSAVASNMRDDEITQDENTVSTSLGHSKDENSKQTCIRIGTKTPKSDSCTPTNTTVEVV<br>GSSGQNGDEKQNIVARKIVKMTKQPAKKKPPPSREKKVTRTILAILLAFIITWAPYNVMVLINTFCAPCIP<br>NTVWTIGYWLCYINSTINPACYALCNATFKKTFKHLLMCHYKNIGATR	51.7	MGC clone	BC106742	2.7
CNR1	Class A	MKSILDGLADTTFRTITTDLLYVGSNDIQYEDIKGDMASKLGYFPQKFPLTSFRGSPFQEKMTAGDNPQ<br>LVPADQVNITEFYNKSLSSFKENEENIQCGENFMDIECFMVLNPSQQLAIAVLSLTLGTFTVLENLLVLC<br>VILHSRSLRCRPSYHFIGSLAVADLLGSVIFVYSFIDFHVFHRKDSRNVFLFKLGGVTASFTASVGSLFLT<br>AIDRYISIHRPLAYKRIVTRPKAVVAFCLMWTIAIVIAVLPLLGWNCEKLQSVCSDIFPHIDETYLMFWIGV<br>TSVLLLFIVYAYMYILWKAHSHAVRMIQRGTQKSIIIHTSEDGKVQVTRPDQARMDIRLAKTLVLILVVLIIC<br>WGPLLAIMVYDVFGKMNKLIKTVFAFCSMLCLLNSTVNPIIYALRSKDLRHAFRSMFPSCEGTAQPLDN<br>SMGDSDCLHKHANNAASVHRAAESCIKSTVKIAKVTMSVSTDTSAEAL	52.9	MGC clone	BC074812	8.5
CXCR4	Class A	MEGISIYTSDNYTEEMGSGDYDSMKEPCFREENANFNKIFLPTIYSIIFLTGIVGNGLVILVMGYQKKLRS<br>MTDKYRLHLSVADLLFVITLPFWAVDAVANWYFGNFLCKAVHVIYTVNLYSSVLILAFISLDRYLAIVHAT<br>NSQRPRKLLAEKVVYVGVWIPALLLTIPDFIFANVSEADDRYICDRFYPNDLWVVVFQFQHIMVGLILPGI<br>VILSCYCIIISKLSHSKGHQKRKALKTTVILILAFFACWLPYYIGISIDSFILLEIIKQGCEFENTVHKWISITE<br>ALAFFHCCLNPILYAFLGAKFKTSAQHALTSVSRGSSLKILSKGKRGGHSSVSTESESSSFHSS	39.7	MGC clone	BC020968	5.2
DRD1	Class A	MRTLNTSAMDGTGLVVERDFSVRILTACFLSLLILSTLLGNTLVCAAVIRFRHLRSKVTNFFVISLAVSDLL<br>VAVLVMPWKAVAEIAGFWPFGSFCNIWVAFDIMCSTASILNLCVISVDRYWAISSPFRYERKMTPKAAFI<br>LISVAWTLSVLISFIPVQLSWHKAKPTSPSDGNATSLAETIDNCDSSLSRTYAISSSVISFYIPVAIMIVTYT<br>RIYRIAQKQIRRIAALERAAVHAKNCQTTTGNGKPVECSQPESSFKMSFKRETKVLKTLSVIMGVFVCC<br>WLPFFILNCILPFCGSGETQPFCIDSNTFDVFVWFGWANSSLNPIIYAFNADFRKAFSTLLGCYRLCPAT<br>NNAIETVSINNNGAAMFSSHHEPRGSISKECNLVYLIPHAVGSSEDLKKEEAAGIARPLEKLSPALSVILD<br>YDTDVSLEKIQPITQNGQHPT	49.3	MGC clone	BC074978	3.2
GHSR	Class A	MWNATPSEEPGFNLTLADLDWDASPGNDSLGDELLQLFPAPLLAGVTATCVALFVVGIAGNLLTMLVV<br>SRFRELRTTTNLYLSSMAFSDLLIFLCMPLDLVRLWQYRPWNFGDLLCKLFQFVSESCTYATVLTITALS<br>VERYFAICFPLRAKVVVTKGRVKLVIFVIWAVAFCSAGPIFVLVGVEHENGTDPWDTNECRPTEFAVRS<br>GLLTVMVWVSSIFFFLPVFCLTVLYSLIGRKLWRRRRGDAVVGASLRDQNHKQTVKMLAVVVFAFILCW<br>LPFHVGRYLFSKSFEPGSLEIAQISQYCNLVSFVLFYLSAAINPILYNIMSKKYRVAVFRLLGFEPFSQRK<br>LSTLKDESSRAWTESSINT	41.3	MGC clone	BC113547	7.3
HRH2	Class A	MAPNGTASSFCLDSTACKITITVVLAVLILITVAGNVVVCLAVGLNRRLRNLTNCFIVSLAITDLLLGLLVLP<br>FSAIYQLSCKWSFGKVFCNIYTSLDVMLCTASILNLFMISLDRYCAVMDPLRYPVLVTPVRVAISLVLIWVI<br>SITLSFLSIHLGWNSRNETSKGNHTTSKCKVQVNEVYGLVDGLVTFYLPLLIMCITYYRIFKVARDQAKRI<br>NHISSWKAATIREHKATVTLAAVMGAFIICWFPYFTAFVYRGLRGDDAINEVLEAIVLWLGYANSALNPIL<br>YAALNRDFRTGYQQLFCCRLANRNSHKTSLRSNASQLSRTQSREPRQQEEKPLKLQVWSGTEVTAP<br>QGATDRPWLCLPECWSVELTHSFIHLFIHSFANIHPIPTTCQEL	44.5	MGC clone	BC054510	5.1
LGR5	Class A	MDTSRLGVLLSLPVLLQLATGGSSPRSGVLLRGCPTHCHCEPDGRMLLRVDCSDLGLSELPSNLSVF<br>TSYLDLSMNNISQLLPNPLPSLRFLEELRLAGNALTYIPKGAFTGLYSLKVLMLQNNQLRHVPTEALQNL<br>RSLQSLRLDANHISYVPPSCFSGLHSLRHLWLDDNALTEIPVQAFRSLSALQAMTLALNKIHHIPDYAFG<br>NLSSLVVLHLHNNRIHSLGKKCFDGLHSLETLDLNYNNLDEFPTAIRTLSNLKELGFHSNNIRSIPEKAFV<br>GNPSLITIHFYDNPIQFVGRSAFQHLPELRTLTLNGASQITEFPDLTGTANLESLTLTGAQISSLPQTVCN<br>QLPNLQVLDLSYNLLEDLPSFSVCQKLQKIDLRHNEIYEIKVDTFQQLLSLRSLNLAWNKIAIIHPNAFST<br>LPSLIKLDLSSNLLSSFPITGLHGLTHLKLTGNHALQSLISSENFPELKVIEMPYAYQCCAFGVCENAYKI<br>SNQWNKGDNSSMDDLHKKDAGMFQAQDERDLEDFLLDFEEDLKALHSVQCSPSPGPFKPCEHLLD<br>GWLIRIGVWTIAVLALTCNALVTSTVFRSPLYISPIKLLIGVIAAVNMLTGVSSAVLAGVDAFTFGSFARHG<br>AWWENGVGCHVIGFLSIFASESSVFLLTLAALERGFSAKYSAKFETKAPFSSLKVIILLCALLALTMAAVP<br>LLGGSKYGASPLCLPLPFGEPSTMGYMVALILLNSLCFLMMTIAYTKLYCNLDKGDLENIWDCSMVKHI<br>ALLLFTNCILNCPVAFLSFSSLINLTFISPEVIKFILLVVVPLPACLNPLLYILFNPHFKEDLVSLRKQTYVWT<br>RSKHPSLMSINSDDVEKQSCDSTQALVTFTSSSITYDLPPSSVPSPAYPVTESCHLSSVAFVPCL	100.0	MGC clone	BC096324	0.8
MCHR1	Class A	MSVGAMKKGVGRAVGLGGGSGCQATEEDPLPDCGACAPGQGGRRWRLPQPAWVEGSSARLWEQ<br>ATGTGWMDLEASLLPTGPNASNTSDGPDNLTSAGSPPRTGSISYINIIMPSVFGTICLLGIIGNSTVIFAV<br>VKKSKLHWCNNVPDIFIINLSVVDLLFLLGMPFMIHQLMGNGVWHFGETMCTLITAMDANSQFTSTYILT<br>AMAIDRYLATVHPISSTKFRKPSVATLVICLLWALSFISITPVWLYARLIPFPGGAVGCGIRLPNPDTDLYW<br>FTLYQFFLAFALPFVVITAAYVRILQRMTSSVAPASQRSIRLRTKRVTRTAIAICLVFFVCWAPYYVLQLTQ<br>LSISRPTLTFVYLYNAAISLGYANSCLNPFVYIVLCETFRKRLVLSVKPAAQGQLRAVSNAQTADEERTE<br>SKGT	46.0	Flexi clone	NA	5.5
MRGPRX1	Class A	MDPTISTLDTELTPINGTEETLCYKQTLSLTVLTCIVSLVGLTGNAVVLWLLGCRMRRNAFSIYILNLAAA<br>DFLFLSGRLIYSLLSFISIPHTISKILYPVMMFSYFAGLSFLSAVSTERCLSVLWPIWYRCHRPTHLSAVVC<br>VLLWALSLLRSILEWMLCGFLFSGADSAWCQTSDFITVAWLIFLCVVLCGSSLVLLIRILCGSRKIPLTRL<br>YVTILLTVLVFLLCGLPFGIQFFLFLWIHVDREVLFCHVHLVSIFLSALNSSANPIIYFFVGSFRQRQNRQN<br>LKLVLQRALQDASEVDEGGGQLPEEILELSGSRLEQ	36.3	Flexi clone	NA	4.2
OR1D2	Class A	MDGGNQSEGSEFLLLGMSESPEQQRILFWMFLSMYLVTVVGNVLIILAISSDSRLHTPVYFFLANLSFT<br>DLFFVTNTIPKMLVNLQSHNKAISYAGCLTQLYFLVSLVALDNLILAVMAYDRYVAICCPLHYTTAMSPKL<br>CILLLSLCWVLSVLYGLIHTLLMTRVTFCGSRKIHYIFCEMYVLLRMACSNIQINHTVLIATGCFIFLIPFGF<br>VIISYVLIIRAILRIPSVSKKYKAFSTCASHLGAVSLFYGTLCMVYLKPLHTYSVKDSVATVMYAVVTPMM<br>NPFIYSLRNKDMHGALGRLLDKHFKRLT	35.2	MGC clone	BC106735	4.7
P2RY2	Class A	MAADLGPWNDTINGTWDGDELGYRCRFNEDFKYVLLPVSYGVVCVLGLCLNAVALYIFLCRLKTWNA<br>STTYMFHLAVSDALYAASLPLLVYYYARGDHWPFSTVLCKLVRFLFYTNLYCSILFLTCISVHRCLGVLR<br>PLRSLRWGRARYARRVAGAVWVLVLACQAPVLYFVTTSARGGRVTCHDTSAPELFSRFVAYSSVMLG<br>LLFAVPFAVILVCYVLMARRLLKPAYGTSGGLPRAKRKSVRTIAVVLAVFALCFLPFHVTRTLYYSFRSLD<br>LSCHTLNAINMAYKVTRPLASANSCLDPVLYFLAGQRLVRFARDAKPPTGPSPATPARRRLGLRRSDR<br>TDMQRIEDVLGSSEDSRRTESTPAGSENTKDIRL	42.3	MGC clone	BC028135	5.3

Symbol	GPCR class	Amino acid sequence	MW (kDa)	Resource[§]	Accession	Productivity in bilayer-dialysis method (mg/ mL wheat germ extract)
RHO	Class A	MNGTEGPNFYVPFSNATGVVRSPFEYPQYYLAEPWQFSMLAAYMFLLIVLGFPINFLTLYVTVQHKKL<br>RTPLNYILLNLAVADLFMVLGGFTSTLYTSLHGYFVFGPTGCNLEGFFATLGGEIALWSLVVLAIERYVVV<br>CKPMSNFRFGENHAIMGVAFTWVMALACAAPPLAGWSRYIPEGLQCSCGIDYYTLKPEVNNESFVIY<br>MFVVHFTIPMIIIFFCYGQLVFTVKEAAAQQQESATTQKAEKEVTRMVIIMVIAFLICWVPYASVAFYIFTH<br>QGSNFGPIFMTIPAFFAKSAAIYNPVIYIMMNKQFRNCMLTTICCGKNPLGDDEASATVSKTETSQVAPA	38.9	MGC clone	BC112104	5.9
BAI1	Class B	MRGQAAAPGPVWILAPLLLLLLLLGRRARAAAGADAGPGPEPCATLVQGKFFGYFSAAAVFPANASRC<br>SWTLRNPDPRRYTLYMKVAKAPVPCSGPGRVRTYQFDSFLESTRTYLGVESFDEVLRLCDPSAPLAF<br>LQASKQFLQMRRQQPPQHDGLRPRAGPPGPTDDFSVEYLVVGNRNPSRAACQMLCRWLDACLAGS<br>RSSHPCGIMQTPCACLGGEAGGPAAGPLAPRGDVCLRDAVAGGPENCLTSLTQDRGGHGATGGWKL<br>WSLWGECTRDCGGGLQTRTRTCLPAPGVEGGGCEGVLEEGRQCNREACGPAGRTSSRSQSLRSTD<br>ARRREELGDELQQFGFPAPQTGDPAAEEWSPWSVCSSTCGEGWQTRTRFCVSSSYSTQCSGPLRE<br>QRLCNNSAVCPVHGAWDEWSPWSLCSSTCGRGFRDRTRTCRPPQFGGNPCEGPEKQTKFCNIALC<br>PGRAVDGNWNEWSSWSACSASCSQGRQQRTRECNGPSYGGAECQGHWVETRDCFLQQCPVDGK<br>WQAWASWGSCSVTCGAGSQRRERVCSGPFFGGAACQGPQDEYRQCGTQRCPEPHEICDEDNFGA<br>VIWKETPAGEVAAVRCPRNATGLILRRCELDEEGIAYWEPPTYIRCVSIDYRNIQMMTREHLAKAQRGL<br>PGEGVSEVIQTLVEISQDGTSYSGDLLSTIDVLRNMTEIFRRAYYSPTPGDVQNFVQILSNLLAEENRDK<br>WEEAQLAGPNAKELFRLVEDFVDVIGFRMKDLRDAYQVTDNLVLSIHKLPASGATDISFPMKGWRATG<br>DWAKVPEDRVTVSKSVFSTGLTEADEASVFVVGTVLYRNLGSFLALQRNTTVLNSKVISVTVKPPPRS<br>LRTPLEIEFAHMYNGTTNQTCILWDETDVPSSSAPPQLGPWSWRGCRTVPLDALRTRCLCDRLSTFAI<br>LAQLSADANMEKATLPSVTLIVGCGVSSLTLLMLVIIYVSVWRYIRSERSVILINFCLSIISSNALILIGQTQT<br>RNKVVCTLVAAFLHFFFLSSFCWVLTEAWQSYMAVTGHLRNRLIRKRFLCLGWGLPALVVAISVGFTKA<br>KGYSTMNYCWLSLEGGLLYAFVGPAAAVVLVNMVIGILVFNKLVSKDGITDKKLKERAGASLWSSCVVL<br>PLLALTWMSAVLAVTDRRSALFQILFAVFDSLEGFVIVMVHCILRREVQDAVKCRVVDRQEEGNGDSG<br>GSFQNGHAQLMTDFEKDVDLACRSVLNKDIAACRTATITGTLKRPSLPEEEKLKLAHAKGPPTNFNSL<br>PANVSKLHLHGSPRYPGGPLPDFPNHSLTLKRDKAPKSSFVGDGDIFKKLDSELSRAQEKALDTSYVIL<br>PTATATLRPKPKEEPKYSIHIDQMPQTRLIHLSTAPEASLPARSPPSRQPPSGGPPEAPPAQPPPPPPP<br>PPPPPQQPLPPPPNLEPAPPSLGDPGEPAAHPGPSTGPSTKNENVATLSVSSLERRKSRYAELDFEKI<br>MHTRKRHQDMFQDLNRKLQHAAEKDKEVLGPDSKPEKQQTPNKRPWESLRKAHGTPTWVKKELEP<br>LQPSPLELRSVEWERSGATIPLVGQDIIDLQTEV	173.5	Flexi clone	NA	1.1
GHRHR	Class B	MDRRMWGAHVFCVLSPLPTVLGHMHPECDFITQLREDESACLQAAEEMPNTTLGCPATWDGLLCWP<br>TAGSGEWVTLPCPDFFSHFSSESGAVKRDCTITGWSEPFPPYPVACPVPLELLAEEESYFSTVKIIYTV<br>GHSISIVALFVAITILVALRRLHCPRNYVHTQLFTTFILKAGAVFLKDAALFHSDDTDHCSFSTVLCKVSVA<br>ASHFATMTNFSWLLAEAVYLNCLLASTSPSSRRAFWWLVLAGWGLPVLFTGTWVSCKLAFEDIACWD<br>LDDTSPYWWIIKGPIVLSVGVNFGLFLNIIRILVRKLEPAQGSLHTQSQYWRLSKSTLFLIPLFGIHYIIFNF<br>LPDNAGLGIRLPLELGLGSFQGFIVAILYCFLNQEVRTEISRKWHGHDPELLPAWRTRAKWTTPSRSAA<br>KVLTSMC	47.4	Flexi clone	NA	7.3
LPHN1	Class B	MARLAAVLWNLCVTAVLVTSATQGLSRAGLPFGLMRRELACEGYPIELRCPGSDVIMVENANYGRTDD<br>KICDADPFQMENVQCYLPDAFKIMSQRCNNRTQCVVVAGSDAFPDPCPGTYKYLEVQYDCVPYKVE<br>QKVFVCPGTLQKVLEPTSTHESEHQSGAWCKDPLQAGDRIYVMPWIPYRTDTLTEYASWEDYVAARH<br>TTTYRLPNRVDGTGFVVYDGAVFYNKERTRNIVKYDLRTRIKSGETVINTANYHDTSPYRWGGKTDIDL<br>AVDENGLWVIYATEGNNGRLVVSQLNPYTLRFEGTWETGYDKRSASNAFMVCGVLYVLRSVYVDDDS<br>EAAGNRVDYAFNTNANREEPVSLTFPNPYQFISSVDYNPRDNQLYVWNNYFVVRYSLEFGPPDPSAG<br>PATSPPLSTTTTARPTPLTSTASPAATTPLRRAPLTTHPVGAINQLGPDLPPATAPVPSTRRPPAPNLHV<br>SPELFCEPREVRRVQWPATQQGMLVERPCPKGTRGIASFQCLPALGLWNPRGPDLSNCTSPWVNQV<br>AQKIKSGENAANIASELARHTRGSIYAGDVSSSVKLMEQLLDILDAQLQALRPIERESAGKNYNKMHKR<br>ERTCKDYIKAVVETVDNLLRPEALESWKDMNATEQVHTATMLLDVLEEGAFLLADNVREPARFLAAKE<br>NVVLEVTVLNTEGQVQELVFPQEEYPRKNSIQLSAKTIKQNSRNGVVKVVFILYNNLGLFLSTENATVKL<br>AGEAGPGGPGGASLVVNSQVIAASINKESSRVFLMDPVIFTVAHLEDKNHFNANCSFWNYSERSMLG<br>YWSTQGCRLVESNKTHTTCACSHLTNFAVLMAHREIYQGRINELLLSVITWVGIVISLVCLAICISTFCFL<br>RGLQTDRNTIHKNLCINLFLAELLFLVGIDKTQYEIACPIFAGLLHYFFLAAFSWLCLEGVHLYLLLVEVFE<br>SEYSRTKYYYLGGYCFPALVVGIAAAIDYRSYGTEKACWLRVDNYFIWSFIGPVSFVIVVNLVFLMVTLH<br>KMIRSSSVLKPDSSRLDNIKSWALGAIALLFLLGLTWAFGLLFINKESVVMAYLFTTFNAFQGVFIFVFHC<br>ALQKKVHKEYSKCLRHSYCCIRSPPGGTHGSLKTSAMRSNTRYYTGTQSRIRRMWNDTVRKQTESS<br>FMAGDINSTPTLNRGTMGNHLLTNPVLQPRGGTSPYNTLIAESVGFNPSSPPVFNSPGSYREPKHPL<br>GGREACGMDTLPLNGNFNNSYSLRSGDFPPGDGGPEPPRGRNLADAAAFEKMIISELVHNNLRGSS<br>SAAKGPPPPEPPVPPVPGGGGEEEAGGPGGADRAEIELLYKALEEPLLLPRAQSVLYQSDLDESESC<br>TAEDGATSRPLSSPPGRDSLYASGANLRDSPSYPDSSPEGPSEALPPPPPAPPGPPEIYYTSRPPALV<br>ARNPLQGYYQVRRPSHEGYLAAPGLEGPGPDGDGQMQLVTSL	162.7	Flexi clone	NA	0.4
PTH1R	Class B	MGTARIAPGLALLLCCPVLSSAYALVDADDVMTKEEQIFLLHRAQAQCEKRLKEVLQRPASIMESDKG<br>WTSASTSGKPRKDKASGKLYPESEEDKEAPTGSRYRGRPCLPEWDHILCWPLGAPGEVVAVPCPDYI<br>YDFNHKGHAYRRCDRNGSWELVPGHNRTWANYSECVKFLTNETREREVFDRLGMIYTVGYSVSLAS<br>LTVAVLILAYFRRLHCTRNYIHMHLFLSFMLRAVSIFVKDAVLYSGATLDEAERLTEEELRAIAQAPPPPAT<br>AAAGYAGCRVAVTFFLYFLATNYYWILVEGLYLHSLIFMAFFSEKKYLWGFTVFGWGLPAVFVAVWVSV<br>RATLANTGCWDLSSGNKKWIIQVPILASIVLNFILFINIVRVLATKLRETNAGRCDTRQQYRKLLKSTLVL<br>MPLFGVHYIVFMATPYTEVSGTLWQVQMHYEMLFNSFQGFFVAIIYCFCNGEVQAEIKKSWSRWTLAL<br>DFKRKARSGSSSYSYGPMVSHTSVTNVGPRVGLGLPLSPRLLPTATTNGHPQLPGHAKPGTPALETL<br>ETTPPAMAAPKDDGFLNGSCSGLDEEASGPERPPALLQEEWETVM	66.4	MGC clone	BC112247	7.2
GABBR1	Class C	MGPGAPFARVGWPLPLLVVMAAGVAPVWASHSPHLPRPHSRVPPHPSSERRAVYIGALFPMSGGWP<br>GGQACQPAVEMALEDVNSRRDILPDYELKLIHHDSKCDPGQATKYLYELLYNDPIKIILMPGCSSVSTLV<br>AEAARMWNLIVLSYGSSSPALSNRQRFPTFFRTHPSATLHNPTRVKLFEKWGWKKIATIQQTTEVFTS<br>TLDDLEERVKEAGIEITFRQSFFSDPAVPVKNLKRQDARIIVGLFYETEARKVFCEVYKERLFGKKYVWF<br>LIGWYADNWFKIYDPSINCTVDEMTEAVEGHITTEIVMLNPANTRSISNMTSQEFVEKLTKRLKRHPEET<br>GGFQEAPLAYDAIWALALALNKTSGGGGRSGVRLEDFNYNNQTITDQIYRAMNSSSFEGVSGHVVFD<br>ASGSRMAWTLIEQLQGGSYKKIGYYDSTKDDLSWSKTDKWIGGSPPADQTLVIKTFRFLSQKLFISVSV<br>LSSLGIVLAVVCLSFNIYNSHVRYIQNSQPNLNNLTAVGCSLALAAVFPLGLDGYHIGRNQFPFVCQARL<br>WLLGLGFSLGYGSMFTKIWWVHTVFTKKEEKKEWRKTLEPWKLYATVGLLVGMDVLTLAIWQIVDPLH<br>RTIETFAKEEPKEDIDVSILPQLEHCSSRKMNTWLGIFYGYKGLLLLLGIFLAYETKSVSTEKINDHRAVG<br>MAIYNVAVLCLITAPVTMILSSQQDAAFAFASLAIVFSSYITLVVLFVPKMRRLITRGEWQSEAQDTMKTG<br>SSTNNNEEEKSRLLEKENRELEKIIAEKEERVSELRHQLQSRQQLRSRRHPPTPPEPSGGLPRGPPEP<br>PDRLSCDGSRVHLLYK	95.1	MGC clone	BC050532	2.3
FZD7	Class C	MRDPGAAAPLSSLGLCALVLALLGALSAGAGAQPYHGEKGISVPDHGFCQPISIPLCTDIAYNQTILPNL<br>LGHTNQEDAGLEVHQFYPLVKVQCSPELRFFLCSMYAPVCTVLDQAIPPCRSLCERARQGCEALMNK<br>FGFQWPERLRCENFPVHGAGEICVGQNTSDGSGGPGGGPTAYPTAPYLPDLPFTALPPGASDGRGR<br>PAFPFSCPRQLKVPPYLGYRFLGERDCGAPCEPGRANGLMYFKEEERRFARLWVGVWSVLCCASTL<br>FTVLTYLVDMRRFSYPERPIIFLSGCYFMVAVAHVAGFLLEDRAVCVERFSDDGYRTVAQGTKKEGCTI<br>LFMVLYFFGMASSIWWVILSLTWFLAAGMKWGHEAIEANSQYFHLAAWAVPAVKTITILAMGQVDGDLL<br>SGVCYVGLSSVDALRGFVLAPLFVYLFIGTSFLLAGFVSLFRIRTIMKHDGTKTEKLEKLMVRIGVFSVL<br>YTVPATIVLACYFYEQAFREHWERTWLLQTCKSYAVPCPPGHFPPMSPDFTVFMIKYLMTMIVGITTGF<br>WIWSGKTLQSWRRFYHRLSHSSKGETAV	63.6	MGC clone	BC015915	3.3
SMO	Class C	MAAARPARGPELPLLGLLLLLLLGDPGRGAASSGNATGPGPRSAGGSARRSAAVTGPPPPLSHCGRA<br>APCEPLRYNVCLGSVLPYGATSTLLAGDSDSQEEAHGKLVLWSGLRNAPRCWAVIQPLLCAVYMPKC<br>ENDRVELPSRTLCQATRGPCAIVERERGWPDFLRCTPDRFPEGCTNEVQNIKFNSSGQCEVPLVRTD<br>NPKSWYEDVEGCGIQCQNPLFTEAEHQDMHSYIAAFGAVTGLCTLFTLATFVADWRNSNRYPAVILFY<br>VNACFFVGSIGWLAQFMDGARREIVCRADGTMRLGEPTSNETLSCVIIFVIVYYALMAGVVWFVVLTYA<br>WHTSFKALGTTYQPLSGKTSYFHLLTWSLPFVLTVAILAVAQVDGDSVSGICFVGYKNYRYRAGFVLAP<br>IGLVLIVGGYFLIRGVMTLFSIKSNHPGLLSEKAASKINETMLRLGIFGFLAFGFVLITFSCHFYDFFNQAE<br>WERSFRDYVLCQANVTIGLPTKQPIPDCEIKNRPSLLVEKINLFAMFGTGIAMSTWVWTKATLLIWRRT<br>WCRLTGQSDDEPKRIKKSKMIAKAFSKRHELLQNPGQELSFSMHTVSHDGPVAGLAFDLNEPSADVS<br>SAWAQHVTKMVARRGAILPQDISVTPVATPVPPEEQANLWLVEAEISPELQKRLGRKKKRRKRKKEVC<br>PLAPPPELHPPAPAPSTIPRLPQLPRQKCLVAAGAWGAGDSCRQGAWTLVSNPFCPEPSPPQDPFLP<br>SAPAPVAWAHGRRQGLGPIHSRTNLMDTELMDADSDF	86.4	MGC clone	BC009989	1.9

Symbol	GPCR class	Amino acid sequence	MW (kDa)	Resource[§]	Accession	Productivity in bilayer-dialysis method (mg/ mL wheat germ extract)
TAS1R1	Class C	MLLCTARLVGLQLLISCCWAFACHSTESSPDFTLPGDYLLAGLFPLHSGCLQVRHRPEVTLCDRSCSF<br>NEHGYHLFQAMRLGVEEINNSTALLPNITLGYQLYDVCSDSANVYATLRVLSLPGQHHIELQGDLLHYS<br>PTVLAVIGPDSTNRAATTAALLSPFLVPMLLEQIHKVHFLLHKDTVAFNDNRDPLSSYNIIAWDWNGPK<br>WTFTVLGSSTWSPVQLNINETKIQWHGKDNQVPKSVCSSDCLEGHQRVVTGFHHCCFECVPCGAGT<br>FLNKSDLYRCQPCGKEEWAPEGSQTCFPRTVVFLALREHTSWVLLAANTLLLLLLLGTAGLFAWHLDT<br>PVVRSAGGRLCFLMLGSLAAGSGSLYGFFGEPTRPACLLRQALFALGFTIFLSCLTVRSFQLIIIFKFSTK<br>VPTFYHAWVQNHGAGLFVMISSAAQLLICLTWLVVWTPLPAREYQRFPHLVMLECTETNSLGFILAFLY<br>NGLLSISAFACSYLGKDLPENYNEAKCVTFSLLFNFVSWIAFFTTASVYDGKYLPAANMMAGLSSLSSG<br>FGGYFLPKCYVILCRPDLNSTEHFQASIQDYTRRCGST	65.1	Flexi clone	NA	4.7
TAS2R14	Class C	MGGVIKSIFTFVLIVEFIIGNLGNSFIALVNCIDWVKGRKISSVDRILTALAISRISLVWLIFGSWCVSVFFPA<br>LFATEKMFRMLTNIWTVINHFSVWLATGLGTFYFLKIANFSNSIFLYLKWRVKKVVLVLLLVTSVFLFLNIA<br>LINIHINASINGYRRNKTCSSDSSNFTRFSSLIVLTSTVFIFIPFTLSLAMFLLLIFSMWKHRKKMQHTVKIS<br>GDASTKAHRGVKSVITFFLLYAIFSLSFFISVWTSERLEENLIILSQVMGMAYPSCHSCVLILGNKKLRQA<br>SLSVLLWLRYMFKDGEPSGHKEFRESS	36.2	MGC clone	BC103699	1.7
VN1R2	Class C	MTHTLYPTPFALYPINISAAWHLGPLPVSCFVSNKYQRSLAFGATTGLRVLVVVVPQTQLSFLSSLCLVS<br>LFLHSLVSAHGEKPTKPVGLDPTLFQVVVGILGNFSLLYYYMFLYFRGYKPRSTDLILRHLTVADSLVILS<br>KRIPETMATFGLKHFDNYFGCKFLLYAHRVGRGVSIGSTCLLSVFQVITINPRNSRWAEMKVKAPTYIGL<br>SNILCWAFHMLVNAIFPIYTTGKWSNNNITKKGDLGYCSAPLSDEVTKSVYAALTSFHDVLCLGLMLWA<br>SSSIVLVLYRHKQQVQHICRNNLYPNSSPGNRAIQSILALVSTFALCYALSFITYVYLALFDNSSWWLVNT<br>AALIIACFPTISPFVLMCRDPSRSRLCSICCRRNRRFFHDFRKM	44.5	MGC clone	BC130356	0.8

NA : Not available

§: Detailed data is abailable from MGC clones (http://mgc.nci.nih.gov/) and Flexi clones (http://www.kazusa.or.jp/kop/), respectively.

Supplementary Table S3. Properties of anti-DRD1 rabbit mAbs obtained in this study

Clone	ELISA	BiLIA	Western blotting	Immuno-precipitation	Affiniy† Kd (nM)	Immuno-staining	Cross-reactivity against mouse Drd1a‡	Epitope region
Ra39	Available	Available	Available	Available	0.45	Available	Cross-react	C-terminus (411-447)
Ra48	Available	Available	Available	Available	0.086	Available	No	C-terminus (374-413)
Ra56	Available	Available	Available	Available	0.54	Available	Cross-react	C-terminus (411-447)
Ra60	Available	Available	Available	Available	0.42	Available	Cross-react	C-terminus (411-447)
Ra62	Available	Available	Available	Available	0.78	Available	No	C-terminus (411-447)
Ra51	Available	Available	Available	Available	0.20	Available	No	Extra cellular roop 2 (196-218)

† : Affinity was determined by scattered plot with ELISA.

‡ : These mAbs did not react with human DRD2, DRD3, DRD5, mouse Drd4 and Drd5.
